# Buffer‐Mediated Catalyst‐Free Strecker Reaction Toward Enzymatic Implementation

**DOI:** 10.1002/open.202500389

**Published:** 2025-09-21

**Authors:** Péter Magyar, Szilárd Újvári, Zsófia Molnár, Zoltán Orgován, Diána Balogh‐Weiser, Blanka Eszter Nagy, Diana Maria Scrob, László Poppe, Péter Ábrányi‐Balogh

**Affiliations:** ^1^ Medicinal Chemistry Research Group HUN‐REN Research Centre for Natural Sciences Magyar tudósok körútja 2 Budapest 1117 Hungary; ^2^ Department of Organic Chemistry and Technology Faculty of Chemical Technology and Biotechnology Budapest University of Technology and Economics Műegyetem rkp. 3 Budapest 1111 Hungary; ^3^ National Drug Research and Development Laboratory HUN‐REN Research Centre for Natural Sciences Magyar tudósok körútja 2 Budapest 1117 Hungary; ^4^ Department of Physical Chemistry and Materials Science Faculty of Chemical Technology and Biotechnology Budapest University of Technology and Economics Műegyetem rkp. 3 Budapest 1111 Hungary; ^5^ Enzymology and Applied Biocatalysis Research Center Faculty of Chemistry and Chemical Engineering Babeş‐Bolyai University of Cluj‐Napoca Arany János str. 11 Cluj‐Napoca 400028 Romania

**Keywords:** aqueous conditions, catalyst‐free, hydroxynitrile lyase, Strecker reaction

## Abstract

A systematic investigation of the catalyst‐free Strecker reaction is conducted in aqueous buffer, offering an efficient and green alternative that leads to α‐aminonitriles without the need of chromatography. Optimization reveals that low pH and high buffer concentration significantly enhance conversion, with yields up to 97%. Broad substrate scope is demonstrated with various aldehydes, ketones, and amines, leading to key intermediates for natural and unnatural amino acids. Potassium cyanide and acetone cyanohydrin are established, latter as a safer and effective cyanide source, and reactions are conducted also in buffer–methyl *tert*‐butyl ether mixed solvent further improving the methodology. It is hypothesized that, based on the similarity to cyanohydrin formation, the hydroxynitrile lyases (HNLs) might transform imines to aminonitriles. Adding AtHNL and HbHNL further accelerates the reaction suggesting an undiscovered reactivity of these enzymes.

## Graphical abstract

Hydroxynitrile lyases (HNLs) are proven to accept imines as substrates and to catalyze the formation of α‐aminonitriles. The process results in the precursors of natural and unnatural α‐amino acids after extraction, without chromatography. This new application of HNL enzymes in Strecker reaction is an example for biocatalysis in multicomponent syntheses.

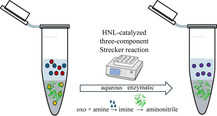

## 1 Introduction

The Strecker reaction was discovered almost 175 years ago in 1851 as the reaction of aldehydes, ammonium chloride, and KCN leading to *α*‐aminonitriles.^[^ ^1^ ^]^ Later, the reaction was applied to diverse ketones and amines, and the scope of the cyanide source was also extended. Since its first discovery, the Strecker reaction was successfully applied for the synthesis precursors of natural and unnatural *α*‐amino acids, alkaloids like saframycin A and girgensohnine, and drugs like vancomycin and clopidogrel.^[^ ^2^ ^–^ ^6^, ^7^ ^]^ The initial inorganic KCN and NaCN were first replaced by trimethylsilyl cyanide that was the most popular reagent (TMSCN), and lately by acetone cyanohydrin and EtOCOCN.^[^ ^3^ ^,^ ^4^ ^,^ ^8^ ^–^ ^10^ ^]^ The reaction is facilitated by acid catalysis, therefore most of the variations include a Lewis‐ or Brønsted acid catalyst or additive. A dozen Lewis acids as ZnCl_2_, AlCl_3_, InCl_3_, Ga(OTf)_3_, or Bi(NO_3_)_3_ can be mentioned, but several salts from d‐block metals (Zr, Ru, Pr, Cu, Sc, La, Gd) were found to be efficient.^[^ ^5^ ^,^ ^8^ ^,^ ^11^, ^12^ ^–^ ^14^ ^]^ Acetic acid is also popular, ionic liquids like [BMIM][ClO_4_], [BMIM][BF_4_], zeolite, sulfonated task‐specific ionic liquid (TSIL), and sulfonic acid modified nanoporous coal or sulfonated nanoreactor was also applied.^[^ ^15^ ^–^ ^18^ ^]^ In most cases, the reaction is carried out in solution, where the solvent might be acetonitrile, toluene, dichloromethane, dichloroethane, or methanol, but almost all organic solvents were described.^[^ ^4^ ^,^ ^5^ ^,^ ^8^ ^,^ ^11^ ^,^ ^19^ ^,^ ^20^ ^]^ A limited number of solventless reactions are also known, usually with a functional material as a catalyst.^[^ ^15^ ^,^ ^21^ ^,^ ^22^ ^]^ Recent sustainable approaches aimed at the use of water as a green and nontoxic solvent.^[^ ^10^ ^,^ ^16^ ^,^ ^17^ ^,^ ^23^ ^,^ ^24^ ^]^ Sustainability, however, is difficult to be reached, as in most cases chromatography is required for the isolation of the products.^[^ ^10^ ^,^ ^15^ ^,^ ^17^ ^,^ ^23^ ^,^ ^24^ ^]^ In the Strecker reaction, the first step is considered to be an imine formation followed by the nucleophilic addition of the cyanide. In case of aldehydes and asymmetric ketones, this results in a racemic mixture. Enantiomeric excess can be achieved by the use of organocatalysts in organic solvents like calix[4]arenes, bifunctional quinine/quinidine‐(thio)urea, or small molecules that can form a complex with the Lewis acid or with sodium or potassium cations and act as a chiral anion generator.^[^ ^2^ ^,^ ^3^ ^,^ ^9^ ^,^ ^13^ ^,^ ^19^ ^,^ ^25^, ^26^, ^27^ ^–^ ^28^ ^]^

Hydroxynitrile lyases (HNLs), also known as oxynitrilases, are responsible for cleaving or building carbon–carbon bonds, particularly in the reversible conversion of mandelonitrile (a cyanohydrin) to hydrogen cyanide and benzaldehyde and, in some cases, show broader substrate promiscuity, including nitroaldolase or esterase activity. First described by Friedrich Wöhler and colleagues in almond species, HNLs are widespread in plants and play a vital role contributing to cyanogenic glycoside metabolism.^[^ ^29^ ^–^ ^31^ ^]^ Despite acting similarly, different types of HNLs (there are two dozen described) are structurally unrelated, highlighting their evolutionary diversity. HNLs are often named after their botanical origin, such as HbHNL from *Hevea brasiliensis* (rubber tree) and AtHNL from *Arabidopsis thaliana* (honeysuckle).^[^ ^30^ ^]^ The biocatalytic potential of HNLs gained research attention in the 1990s, and since then several aliphatic and aromatic ketones were subjected to cyanohydrin formation by HNLs including acetone, benzaldehyde, hex‐2‐ynal, cyclohexyl‐phenyl‐ketones, cyclohexanones, or tetrahydrothiophenone.^[^ ^32^, ^33^, ^34^ ^–^ ^35^ ^]^ Recently, the relationship between the size of the active site, catalytic activity, and promiscuity was investigated.^[^ ^36^ ^,^ ^37^ ^]^ If the active site of the enzyme is larger, several different orientations of the substrates are possible, making the transition state more stable. It was also found that, e.g., LuHNL and XaHNL has a narrow substrate range, PhaHNL favors aromatic and heterocyclic carbonyls rather than aliphatic substrates, and HbHNL, MeHNL catalyze the synthesis of cyanohydrins from a wide range of substrate either aliphatic, aromatic, or heteroaromatic.^[^ ^30^ ^,^ ^38^, ^39^ ^–^ ^40^ ^]^ Notably, the efficiency of the enzyme can be influenced by the reaction medium, the cyanide source, water content, and pH. Aqueous medium and low pH (in the pH 3–6 range), mostly in citrate buffers, facilitates the cyanohydrin formation and the enantioselectivity and biphasic systems are beneficial to enable increased substrate concentration and high product yield.^[^ ^30^ ^,^ ^41^ ^,^ ^42^ ^]^ Among organic solvents, ethers are preferred and the enzyme activity can be obtained rather at the interface than in one phase, and by increasing its polarity in the reaction medium by increasing the buffer concentration (50–500 mM), electrostatic interaction is possible, which further increases the catalytic efficiency of the enzyme.^[^ ^43^, ^44^, ^45^ ^–^ ^46^ ^]^

We decided to optimize a catalyst free Strecker reaction in aqueous medium in that the cyanide addition is promoted by the acidic conditions provided by the buffer. We aimed to isolate the products forming in high conversion by a simple extraction. Having in mind that HNLs are effective in cyanohydrin formation from oxo compounds in citrate buffer and mixed solvents, we aimed to investigate these conditions for the analogous transformation of imines to aminonitriles, and if possible, implement enzymatic catalysis that would be the first proof for an undiscovered reactivity of HNLs.

## 2 Results and Discussion

First, we studied the formation of 2‐(benzylamino)‐2‐phenylacetonitrile (**4aa**) in the reaction of benzaldehyde (**1a**), benzylamine (**2a**), and KCN in citrate buffer at different pHs and buffer concentrations (**Table** **1**). The conversion was followed by HPLC‐MS and ^1^H NMR, and the amount of the formed aminonitrile (**4aa**) or the imine intermediate (**3aa**) was determined from ^1^H NMR (DMSO‐*d*_*6*_) integral of the C—H proton common in the aldehyde (10.1 ppm), imine (8.5 ppm), and aminonitrile (4.76 ppm) highlighted by blue in heading of Table 1. There was no need for chromatography, the products could be isolated by extraction. In water at neutral pH only imine **3aa** formed, but no aminonitrile was detected (Table 1, entry 1). Using 50 mM citrate buffer at pH 5.4 after 1 h low conversion was observed (28%, Table 1, entry 2) that increased slightly after 24 h (39%, Table 1, entry 3). Adding any components in excess did not change the yield significantly. Next, by changing the pH, we observed that the amount of **4aa** aminonitrile increases with the increase of acidity but has a limit of 64% (Table 1 entries 3–7). Increasing the buffer concentration at pH 5.4 improved the conversion (Table 1, entries 8–10), and after 1 h 87% was reached (Table 1, entry 10) that increased to 94% after 24 h (Table 1, entry 11). The decrease of the pH to 5.0 or 3.3 led to 97% conversions (Table 1, entries 12,13).

**Table 1:** Optimization of the reaction of benzaldehyde, benzylamine, and KCN in citrate buffer.

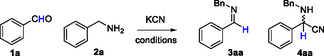					
Entrya)	Reaction time [h]	Buffer concentration [mM]	pH	Imine [**3aa**, %]	Aminonitrile [**4aa**, %]
1b)	1	–	7.0	> 99	0
2	1	50	5.4	72	28
3	24	50		61	39
4			5.0	55	45
5			4.5	52	48
6			3.8	43	57
7			3.3	36	64
8	1	100	5.4	53	47
9		200		37	63
10		500		13	87
11	24			6	94
12	1		5.0	3	97
13			3.3	3	97

a) The reactions were performed in 8 mL volume at 250 mM substrate concentration at room temperature.

b) The reaction was performed in water at pH 7.0.

Next, having these conditions in hand, we extended the scope to a large set of aldehydes and ketones (**Table** **2**). We found that in almost every case the conversion determined by ^1^H NMR was > 90%, and the products were isolated by a simple extraction with good to excellent yields (62%–94%). No significant steric effect was observed, as formaldehyde (**1b**), acetaldehyde (**1c**), acetone (**1d**), benzaldehyde (**1a**), or benzophenone (**1g**) gave very similar conversions (Table 2, entries 1–4,7, respectively). Neither did the substituents of benzaldehyde significantly affect the reaction shown by chloride (**1i‐k**), methyl (**1l**,**m**), methoxy (**1n**,**o**), and cyano (**1p**) substituents (Table 2, entries 9–16). Precursors for glycine (**4ba**), alanine (**4ca**), valine (**4ea**), leucine (**4fa**), and methionine (**4ha**) were successfully synthesized (Table 2, entries 2,3,5,6,8, respectively). These reactions were also performed using acetone cyanohydrin as cyanide source that is considered as a convenient and comparatively safer alternative to KCN,^[^ ^10^ ^,^ ^47^ ^,^ ^48^ ^]^ and in every case a slight increase in the yields was observed. Therefore, in the next investigations acetone cyanohydrin was used as the cyanide precursor.

**Table 2:** Scope of aldehydes and ketones reacting with benzylamine and KCN or acetone cyanohydrin.

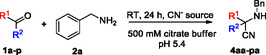								
Entrya)	Aldehyde/Ketone	**R** ^**1**^	**R** ^**2**^	Product	Conversiona)		Isolated yielda)	
1	**1a**	Ph	H	**4aa**	94%		78%	
2	**1b**	H	H	**4ba**	95%	98%b)	94%	97%b)
3	**1c**	Me	H	**4ca**	88%	92%b)	62%	81%b)
4	**1d**	Me	Me	**4da**	99%		92%	
5	**1e**	*i*‐Pr	H	**4ea**	95%	97%b)	76%	83%b)
6	**1f**	2‐MePr	H	**4fa**	94%	95%b)	92%	93%b)
7	**1g**	Ph	Ph	**4ga**	92%		83%	
8	**1h**	‐(CH_2_)_2_‐S‐CH_3_	H	**4ha**	98%	98%b)	94%	96%b)
9	**1i**	2‐Cl‐C_6_H_4_	H	**4ia**	96%		88%	
10	**1j**	3‐Cl‐C_6_H_4_	H	**4ja**	98%		88%	
11	**1k**	4‐Cl‐C_6_H_4_	H	**4ka**	77%		71%	
12	**1l**	3‐Me‐C_6_H_4_	H	**4la**	98%		94%	
13	**1m**	4‐Me‐C_6_H_4_	H	**4ma**	98%		85%	
14	**1n**	2‐OMe‐C_6_H_4_	H	**4na**	95%		94%	
15	**1o**	4‐OMe‐C_6_H_4_	H	**4oa**	96%		90%	
16	**1p**	4‐CN‐C_6_H_4_	H	**4pa**	93%		81%	

a) The reactions were performed in 8 mL volume at 250 mM substrate concentration at room temperature. KCN was used a cyanide source unless noted differently. Conversion was determined by ^1^H NMR;

b) Acetone cyanohydrin used as cyanide source.

**Figure 1:**
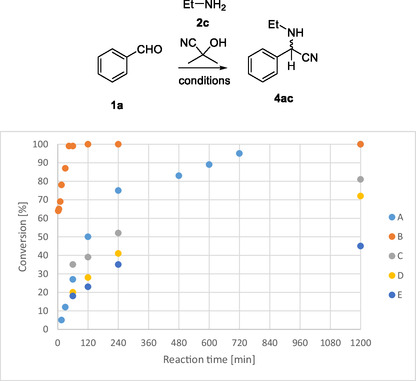
Conversion depending on the buffer and benzaldehyde concentration conditions: A) 50 mM citrate buffer pH 5.4:MTBE 1:1, 250 mM benzaldehyde in 2 mL; B) 500 mM citrate buffer pH 5.4:MTBE 1:1, 250 mM benzaldehyde in 2 mL; C) 500 mM citrate buffer pH 5.4:MTBE 1:1, 100 mM benzaldehyde in 2 mL; D) 500 mM citrate buffer pH 5.4:MTBE 1:1, 50 mM benzaldehyde in 2 mL; and E) 500 mM citrate buffer pH 5.4:MTBE 1:1, 10 mM benzaldehyde in 2 mL. Ethylamine and acetone cyanohydrin was used in 2x excess.

Next, we investigated the effect of a mixed solvent that is used in enzymatic reactions [buffer : methyl *tert*‐butyl ether (MTBE) 1:1]. Bearing in mind that the HNLs might accept smaller substrates, the reaction of benzaldehyde (**1a**) and ethyl amine (**2c**, in 72% aqueous solution) was chosen as a model reaction. We observed first moderate conversion (50%) in the reaction with acetone cyanohydrin in buffer : MTBE 1:1 (500 mM citrate buffer, pH 5.4, 24 h). We increased the yield by using two times excess of **2c** (58%) or acetone cyanohydrin (69%) and reached full conversion (99%) with two times excess of both reagents. Then, we compared 50 mM and 500 mM buffer concentration and measured the conversion at different time points (**Figure** **1** conditions A and B). We found that the buffer with lower concentration requires 20 h for the full conversion (condition B), but increasing the buffer concentration the full conversion was reached after 45 min. Decreasing the substrate concentration to 100, 50, and 10 mM, the conversions after 20 h were 81%, 72%, and 45% (conditions C–E, respectively).

Next, we investigated the scope of amines (**2a‐g**) and benzaldehyde (**1a**) or isobutyrylaldehyde (**1e**) using condition B (**Table** **3**). The latter was chosen to demonstrate that aliphatic aldehydes are also applicable. We reached ≈90% conversion in almost all cases and isolated the aminonitriles in 60%–99% yields. With benzaldehyde methyl‐ (**2b**), ethyl‐ (**2c**), and isopropylamine (**2e**) performed the best (Table 3, entries 2,3,5), but for amines with Pr (**2d**), Bu (**2f**), or Bn (**2a**) substituent longer reaction time was necessary (Table 3, entries 4,6,1). In case of isobutyrylaldehyde (**1e**), methyl‐ (**2b**), ethyl‐ (**2c**), and benzylamine (**2a**) gave the highest yields (Table 3, entries 9,10,8), while propyl‐ (**2d**), *t*‐butyl‐ (**2g**), and butylamine (**2f**) resulted in the lowest yields (Table 3, entries 11,14,13). In general, for propyl‐ (**2d, e**) and butylamines (**2f, g**) the branched isomers (*i‐*Pr (**2e**) and *t*‐Bu (**2g**)) provided better results than the open‐chain derivatives.

**Table 3:** Scope of amines with two aldehydes.

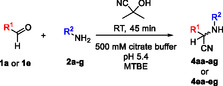					
Entry	**R** ^**1**^	**R** ^**2**^	Product	Conversion	Isolated yield
1	Ph (**1a**)	Bna) (**2a**)	**4aa**	85%	60%
2		Me (**2b**)	**4ab**	95%	91%
3		Et (**2c**)	**4ac**	99%	99%
4		Pra) (**2d**)	**4ad**	90%	77%
5		*i*‐Pr (**2e**)	**4ae**	90%	88%
6		Bua) (**2f**)	**4af**	87%	65%
7		*t*‐Bu (**2g**)	**4ag**	88%	71%
8	*i*‐Pr (**1e**)	Bn (**2a**)	**4ea**	90%	85%
9		Me (**2b**)	**4eb**	90%	85%
10		Et (**2c**)	**4ec**	89%	82%
11		Pr (**2d**)	**4ed**	90%	70%
12		*i*‐Pr (**2e**)	**4ee**	89%	77%
13		Bu (**2f**)	**4ef**	90%	60%
14		*t*‐Bu (**2g**)	**4eg**	90%	68%

a) 20 h reaction time was necessary.

Finally, we investigated whether AtHNL or HbHNL enzymes catalyze the reaction. We chose condition C (aldehydes **1a** and **1e** in 100 mM with two times excess of **2c** and **2a** amines, respectively, and two times excess acetone cyanohydrin) for two reactions, one with isobutyryl aldehyde (**1e**) and benzylamine (**2a**), and one with benzaldehyde (**1a**) and ethylamine (**2c**) (**Table** **4**) in buffer : MTBE 1:1 and 1:4 mixed solvents. First, without enzymes the reactions provided products **4ac** and **4ea** in 30% and 35% yields, respectively, in 1 h (Table 4, entries 1,18). When the enzymes were used in 500 nM concentration, the formation of the aminonitriles increased to 43%–52% (Table 4, entries 2,8,19,25). Adding more enzymes (1 and 2 µM) was beneficial reaching yields up to 75% (Table 4 entries 3,4,9,10,20,21,26,27). Changing the solvent mixture to buffer : MTBE 1:4 in the presence of 2 µM enzymes provided further advantages leading up to 88% yield after 1 h (Table 4, entries 5,6,11,12,22,23,28,29). When the reaction was conducted for 4 h with 2 µM enzyme concentration, a further increase in yield (>92% in all cases, Table 4, entries 7,13,24,30) was still observed that was significant compared to the moderately effective reactions without enzymes (52% and 59%) (Table 4, entries 14,31). In addition, to prove the specific effect of the enzymes, we performed the reactions in the presence of bovine serum albumin (BSA) or myoglobin (Myo) instead of the HNLs. We found only recovered starting amine in the reactions of **1e** and **2a** (Table 4, entries 15,16), while for **1a** and **2c** in the presence of BSA there was no reaction (Table 4, entry 32), and the use of Myo led to 43% product (Table 4, entry 33). Next, to prove that the effect of the increasing amount of enzymes is not caused by possible increasing acidity due to acidic groups, we performed the reactions leading to **4ac** and **4ea** at pH 5.0, but detected only 41% and 42% conversions, respectively (Table 4, entries 17,34). As the final control in citrate buffer, we applied benzaldehyde (**1a**) and benzylamine (**2a**) in the presence of the HNLs. We assumed that due to the larger size, the intermediate and the product would not fit into the enzymes and the conversion would be poor. Indeed, we observed 44% and 28% conversions in the presence of AtHNL and HbHNL, respectively. Finally, we changed the buffer to 500 mM PBS, and investigated the reaction of **1a** and **2c** at pH 6.0, 7.0, and 8.0. In the absence of the biocatalyst, we isolated **4ac** in 21%, 15%, and 10% yields decreasing in parallel with the increasing pH. Adding AtHNL or HbHNL, the highest yields at pH 6.0 were 81% and 80%, respectively, and with the increasing pH, the yields decreased to 60% and 56% at neutral pH and to 43% and 40% at pH 8.0 (Table S1, Supporting Information).

**Table 4:** Effect of HNLs on the reactions of **1e** with **2a** and **1a** with **2c**.

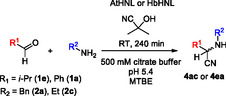						
Entry	R	Reaction time[h]	Solvent mixture buffer: MTBE	Enzyme	Enzyme concentration [µM]	Isolated yield [%]
1	R^1^ = *i*‐Pr R^2^ = Bn	1	1:1	–	–	30
2				AtHNL	0.5	49
3					1	60
4					2	65
5			1:4		1	80
6					2	85
7		4			2	**93**
8		1	1:1	HbHNL	0.5	43
9					1	56
10					2	64
11			1:4		1	78
12					2	82
13		4			2	**92**
14				–	–	52
15				BSA	2	0
16				Myo	2	0
17				–a)	–	41
18	R^1^ = PhR^2^ = Et	1	1:1	–	–	35
19				AtHNL	0.5	52
20					1	70
21					2	75
22			1:4		1	83
23					2	88
24		4			2	**96**
25		1	1:1	HbHNL	0.5	50
26					1	69
27					2	74
28			1:4		1	78
29					2	87
30		4			2	**94**
31				–	–	59
32				BSA	2	0
33				Myo	2	43
34				–a)	–	42

a) using pH 5.0 citrate puffer.

We could thus clearly observe the beneficial effect of the presence of the enzymes and the advantage of having a larger proportion of organic solvent present that might have increased the conversion as well on the amount of the expanding biphasic interface. Although the product formation was enhanced, chiral HPLC indicated that the products of the enzyme mediated process were racemic.

**Figure 2:**
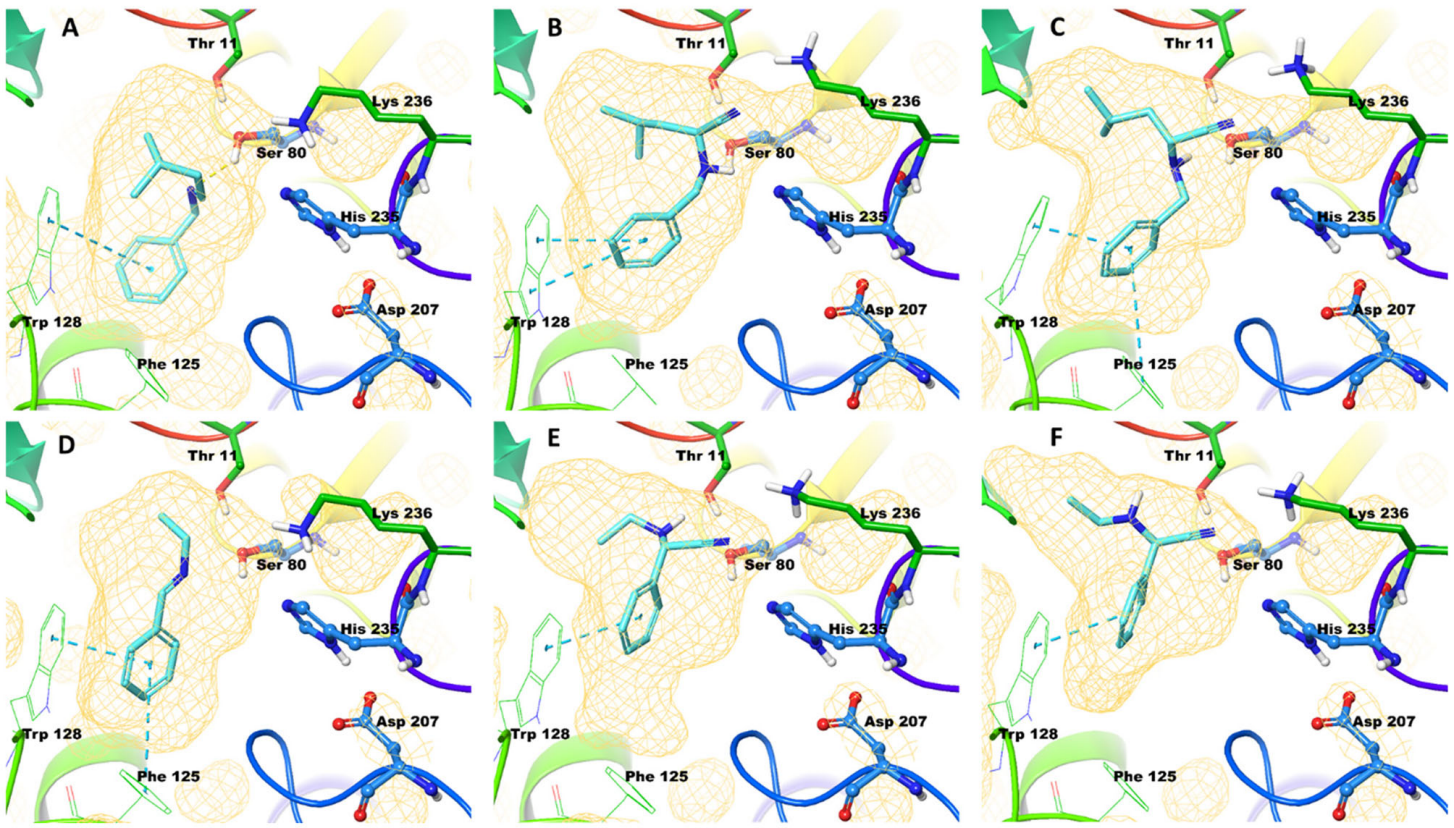
Computed binding modes of A) **3ea**, D) **3ac**, B) *S*‐**4ea**, E) *S*‐**4ac**, C) *R*‐**4ea,** and F) *R*‐**4ac** in HbHNL (PDB 1YB6).

We used computational modeling to explain the observed reactivity enhancement and the lack of stereoselectivity taking the reverse‐Lys mechanism of HbHNL as an example.^[^ ^49^ ^]^ Induced fit docking both enantiomers of products **4ac** and **4ea** into the active site of *S*‐selective HbHNL (PDB 1YB6), we found that the *S*‐**4ea** and *S*‐**4ac** (**Figure** **2B,E**) fits better into the pocket and interacts through the NH with Ser80 or Thr11 and through the CN group with Lys236 similarly to cyanohydrins described in the literature compared to *R‐* **4ea** and *R‐* **4ac** (Figure 2C,F).^[^ ^49^ ^]^ Imines **3ea** and **3ac** fit well to the binding site and interact with amino acids Ser80 and Thr11 similarly to the usual oxo substrates of the enzyme, explaining why HbHNL increase the yield of the reactions (Figure 2A,D). In *S*‐selective HbHNLs, the cyanide addition might take place via the interaction with Lys236. However, we did not find steric hindrance that would influence the addition of the cyanide from the opposite side of the C=N bond. This suggests that although the positioning of the products is more advantageous as expected for the *S*‐aminonitriles, the determining factor for the enantioselectivity might be the lack of the steric hindrance around the imine that would influence the side of the cyanide attack. We hypothesize, therefore, that stereoselectivity could be induced if the opposite side of the imine compared to Lys236 would be sterically closed excluding the cyanide ion from that area.

## 3 Conclusions

In this study, we demonstrated that the Strecker reaction can be performed in aqueous citrate buffer under mild conditions without external catalyst with broad substrate scope and high efficiency. Systematic optimization revealed that citrate buffer at low pH and with high buffer concentration effectively promotes aminonitrile formation, achieving up to 97% yield without the need of chromatography. We also established the reaction's applicability to various aldehydes, ketones, and amines, including precursors for several natural and unnatural *α*‐amino acids. The use of acetone cyanohydrin as a cyanide source further improved safety and product yields and the use of mixed solvents enabled the implementation of biocatalysis. We showed for the first time that HNL enzymes can catalyze the cyanide addition not only to oxo compounds, but imines, as well. Introducing AtHNL and HbHNL enhanced the yields of the reaction compared to the conditions in the absence of the enzyme, although the products remained racemic. Modeling the binding of imines and (*S*) or (*R*) products into HbHNL suggests no steric hindrance for the cyanide addition to the imine, although the (*S*) products fit better to the binding site. Our findings, in general, provide a practical and sustainable synthetic approach to aminonitriles, combining operational simplicity, high efficiency, and enzymatic potential, with applications in synthetic chemistry.

## 4 Experimental Section

4.1

4.1.1

All chemical reagents and solvents were purchased from Fluorochem, Merck/Sigma‐Aldrich, ThermoFisher, BLD, Ambeed, Combi‐Blocks, Enamine, or VWR International and used as received. NMR solvents were purchased from Eurisotop. NMR spectra (^1^H, ^13^C) were recorded at room temperature, on Varian Unity Inova 300 or 500 spectrometers (300 or 500 MHz for ^1^H NMR spectra, 75 or 125 MHz for ^13^C NMR spectra, respectively). All chemical shifts (*δ*) are quoted in parts per million ( ppm), measured from the center of the signal except in the case of multiplets, which are quoted as a range. Coupling constants (*J*) are given with an accuracy of 0.1 Hz. Splitting patterns are abbreviated as follows: singlet (s), doublet (d), triplet (t), quartet (q), multiplet (m), broad singlet (bs), and combinations thereof. HPLC‐MS measurements were performed using a Shimadzu LCMS‐2020 device equipped with a Reprospher 100 C18 (5 μm; 100 x 3 mm) column and positive–negative double ion source (DUIS±) with a quadrupole MS analyzer in a range of 50–1000 m z^−1^. Sample was eluted with gradient elution using eluent A (0.1% formic acid in water) and eluent B (10.1% formic acid in acetonitrile). Flow rate was set to 1 mL min^−1^. The initial condition was 0% B eluent, followed by a linear gradient to 100% B eluent by 1 min, from 1 to 3.5 min 100% B eluent was retained; and from 3.5 to 4.5 min back to initial condition with 5% B eluent and retained to 5 min. The column temperature was kept at room temperature and the injection volume was 1–10 μL. Purity of compounds was assessed by HPLC with UV detection and ^1^H NMR; all tested compounds were > 95% pure, only some solvent traces could be detected in few ^1^H NMR spectra. The enantiomeric excess of the products was determined on a chiral stationary phase HPLC (Chiralcel OJ 250  ×  4.6  mm, 5  µm column) using hexane:*i‐*PrOH = 9:1 as eluent with a flow rate of 1 mL min^−1^, detected at 210 and 254 nm.

##### General Methods for Aminonitrile Synthesis

Method A: Strecker syntheses were carried out in a 20 mL glass vial with a magnetic stirring bar. The aldehyde or ketone (2 mmol) and benzyl amine (2 mmol, 214 mg) were mixed and 8 mL of the buffer was added. Then potassium cyanide (2 mmol, 130 mg) was added and the reaction was stirred at room temperature. After reaching full conversion 15 mL of water was added, and was extracted three times with ethyl acetate (20 mL). The organic phase was dried (Na_2_SO_4_) and concentrated with a rotary vacuum evaporator.

Method B: The oxo compound and 2 equiv. amine was mixed in a 5 mL vial and placed in a shaker. The mixture was shaked at 550 rpm for 10 min at room temperature. Then 2 mL of solvent (buffer, or buffer : MTBE mixture) and 2 equiv. of acetone cyanohydrin was added. After reaching full conversion, 5 mL saturated saline was added and extracted two times with ethyl acetate (10 mL). The organic phase was dried (Na_2_SO_4_) and concentrated with a rotary vacuum evaporator.

##### Compound Characterization

NMR spectra are provided in the Supporting Information.

2‐(Benzylamino)‐2‐phenylacetonitrile (**4aa**)

Yield: **Method A:** 347 mg (78%), yellow oil. ^1^H NMR (300 MHz, CDCl_3_) *δ* 7.61–7.51 (m, 2H) 7.41–7.10 (m, 8H), 4.76 (s, 1H), 4.15–3.90 (m, 2H), 1.94 (bs, 1H) ppm.^[^ ^50^ ^]^

2‐(Benzylamino)acetonitrile (**4ba**)

Yield: Method A 275 mg (94%), yellow oil. Method B 290 mg (99%). ^1^H NMR (300 MHz, CDCl_3_) *δ* 7.16–6.91 (m, 5H), 3.63 (t, *J* = 4.8 Hz, 2H), 3.32–3.21 (m, 2H), 1.56 (s, 1H) ppm.^[^ ^51^ ^]^

2‐(Benzylamino)propanenitrile (**4ca**)

Yield: **Method A:** 197 mg (62%) **Method B:** 270 mg (85%), yellow oil. ^1^H NMR (300 MHz, CDCl_3_) *δ* 7.38–7.27 (m, 5H), 4.07 (d, *J* = 12.9 Hz, 1H), 3.83 (d, *J* = 12.9 Hz, 1H), 3.60 (q, *J* = 7.0 Hz, 1H), 1.58 (bs, 1H), 1.50 (d, *J* = 7.1 Hz, 3H) ppm.^[^ ^52^ ^]^

2‐(Benzylamino)‐2‐methylpropanenitrile (**4da**)

Yield: **Method A:** 320 mg (92%), yellow oil. ^1^H NMR (300 MHz, CDCl_3_) *δ* 7.42–7.25 (m, 5H), 3.91 (s, 2H), 1.53 (s, 6H) ppm.^[^ ^53^ ^]^

2‐(Benzylamino)‐3‐methylbutanenitrile (**4ea**)

Yield: **Method A**: 286 mg (76%), yellow oil. ^1^H NMR (300 MHz, CDCl_3_) *δ* 7.44–7.23 (m, 5H), 4.09 (d, *J* = 13.3 Hz, 1H), 3.83 (d, *J* = 13. 1 Hz, 1H), 3.30 (d, *J* = 6.0 Hz, 1H), 2.08–1.94 (m, 1H), 1.75 (s, 1H), 1.09 (dd, *J* = 6.8, 3.0 Hz, 6H) ppm.^[^ ^54^ ^]^

2‐(Benzylamino)‐4‐methylpentanenitrile **(4fa)**

Yield: **Method A:** 331 mg (82%), orange oil. ^1^H NMR (300 MHz, CDCl_3_) *δ* 7.56–7.14 (m, 5H), 4.08 (d, *J* = 12.8 Hz, 1H), 3.83 (d, *J* = 12.9 Hz, 1H), 3.55 (t, *J* = 7.7 Hz, 1H), 2.17 (s, 1H), 1.90 (td, *J* = 13.7, 7.1 Hz, 1H), 1.66 (t, *J* = 7.1 Hz, 2H), 0.93 (dd, *J* = 6.6, 4.2 Hz, 6H) ppm. ^13^C NMR (75 MHz, CDCl_3_) *δ* 138.44, 128.57, 127.53, 120.46, 51.60, 48.17, 42.42, 24.89, 22.28, 22.12 ppm. HRMS (TOF‐MS): m/z [M + H]^+^ calculated for C_13_H_18_N_2_: 203.1542; found: 203.1548.

2‐(Benzylamino)−2,2‐diphenylacetonitrile (**4ga**)

Yield: **Method B:** 495 mg (83%), yellow oil. ^1^H NMR (300 MHz, CDCl_3_) *δ* 7.58–7.46 (m, 5H), 7.35–7.26 (m, 2H), 7.24–7.14 (m, 5H), 7.07–7.00 (m, 2H), 7.00–6.90 (m, 1H), 3.61–3.54 (m, 2H), 1.19 (s, 1H) ppm.^[^ ^55^ ^]^

2‐(Benzylamino)‐4‐(methylthio)butanenitrile (**4ha**)

Yield: **Method A:** 414 mg (94%), **Method B:** 427 mg (97%), yellow oil. ^1^H NMR (300 MHz, CDCl_3_) *δ* 7.43–7.24 (m, 5H), 4.07 (d, *J* = 12.8 Hz, 1H), 3.83 (d, *J* = 12.8 Hz, 1H), 3.75 (t, *J* = 7.2 Hz, 1H), 2.73–2.66 (m, 2H), 2.11–2.02 (m, 5H) ppm.^13^C NMR (75 MHz, CDCl_3_) *δ* 138.33, 128.73, 128.45, 127.74, 120.03, 51.73, 48.51, 32.89, 30.17, 15.57 ppm. HRMS (TOF‐MS): m/z [M + H]^+^ calculated for C_12_H_16_N_2_S: 221.1112; found: 221.1111.

2‐(Benzylamino)‐2‐(2‐chlorophenyl)acetonitrile (**4ia)**

Yield: **Method A:** 451 mg (88%), yellow oil. ^1^H NMR (300 MHz, CDCl_3_) *δ* 7.66–7.54 (m, 1H), 7.45–7.19 (m, 8H), 5.01 (s, 1H), 4.29–3.66 (m, 2H), 2.19 (s, 1H) ppm.^[^ ^18^ ^]^

2‐(Benzylamino)‐2‐(3‐chlorophenyl)acetonitrile (**4ja**)

Yield: **Method B:** 452 mg (88%), yellow oil. ^1^H NMR (300 MHz, CDCl_3_) *δ* 7.46–7.38 (m, 1H), 7.37–7.08 (m, 8H), 4.66–4.55 (m, 1H), 3.99–3.70 (m, 2H), 2.42 (s, 1H) ppm.^[^ ^56^ ^]^

2‐(Benzylamino)‐2‐(4‐chlorophenyl)acetonitrile (**4ka)**

Yield: **Method A:** 368 mg (71%) **Method B:** 475 mg (92%) orange oil. ^1^H NMR (300 MHz, CDCl_3_) *δ* 7.53–7.46 (m, 2H), 7.41–7.36 (m, 6H), 7.36–7.30 (m, 1H), 4.74 (s, 1H), 4.11–3.86 (m, 2H), 2.32 (s, 1H) ppm.^[^ ^57^ ^]^

2‐(Benzylamino)‐2‐(3‐methylphenyl)acetonitrile (**4la**)

Yield: **Method A:** 444 mg (94%), yellow oil. ^1^H NMR (300 MHz, CDC_3_) *δ* 7.48–7.17 (m, 9H), 4.72 (s, 1H), 4.15–3.90 (m, 2H), 2.39 (s, 3H), 1.98 (s, 1H) ppm.^[^ ^58^ ^]^

2‐(Benzylamino)‐2‐(4‐methylphenyl)acetonitrile (**4ma**)

Yield: **Method A:** 402 mg (85%), yellow oil. ^1^H NMR (300 MHz, CDCl_3_) *δ* 7.49–7.20 (m, 9H), 4.72 (s, 1H), 4.11–3.92 (m, 2H), 2.38 (s, 3H), 2.07 (s, 1H) ppm.^[^ ^58^ ^]^

2‐(Benzylamino)‐2‐(2‐methoxyphenyl)acetonitrile (**4na**)

Yield: **Method A:** 481 mg (94%), yellow oil. ^1^H NMR (300 MHz, CDCl_3_) *δ* 6.65–6.37 (m, 7H), 6.12 (q, J = 9.2 Hz, 2H), 3.99 (s, 1H), 3.35–3.07 (m, 2H), 3.03 (s, 3H), 1.43 (s, 1H) ppm.^[^ ^59^ ^]^

2‐(Benzylamino)‐2‐(4‐methoxyphenyl)acetonitrile (**4oa**)

Yield: **Method A:** 454 mg (90%), yellow oil. ^1^H NMR (300 MHz, CDCl_3_) *δ* 7.50–7.26 (m, 7H), 6.93 (d, J = 8.7 Hz, 2H), 4.70 (s, 1H), 4.11–3.87 (m, 2H), 3.82 (s, 3H), 2.17 (s, 1H) ppm.^[^ ^58^ ^]^

4‐((Benzylamino)(cyano)methyl)benzonitrile **(4 pa).**

Yield: **Method A:** 400 mg (81%), yellow oil. ^1^H NMR (300 MHz, CDCl_3_) *δ* 7.71 (s, 4H), 7.43–7.30 (m, 5H), 4.82 (s, 1H), 4.12–3.85 (m, 2H), 1.98 (s, 1H) ppm; ^13^C NMR (75 MHz, CDCl_3_) *δ* 134.96, 132.76, 127.97, 124.02, 123.68, 123.38, 123.18, 113.35, 112.95, 108.40, 48.22, 46.52 ppm. HRMS (TOF‐MS): m/z [M + H]^+^ calculated for C_16_H_13_N_3_: 248.1187; found: 248.1187.

2‐(Methylamino)‐2‐phenylacetonitrile (**4ab**)

Yield: **Method A:** 50 mg (68%) **Method B:** 67 mg (91%), yellow oil. ^1^H NMR (300 MHz, CDCl_3_) *δ* 7.61–7.48 (m, 2H), 7.46–7.34 (m, 3H), 4.76 (s, 1H), 2.58 (s, 3H) ppm.^[^ ^60^ ^]^

2‐(Ethylamino)‐2‐phenylacetonitrile (**4ac**)

Yield: **Method A:** 50 mg (62%) **Method B:** 79 mg (99%), brownish‐yellow oil. ^1^H NMR (300 MHz, CDCl_3_) *δ* 7.58–7.46 (m, 2H), 7.46–7.33 (m, 3H), 4.80 (s, 1H), 3.00–2.70 (m, 2H), 1.60 (bs, 1H), 1.17 (t, *J* = 7.1 Hz, 3H) ppm.^[^ ^61^ ^]^

2‐Phenyl‐2‐(propylamino)acetonitrile (**4ad**)

Yield: **Method B:** 67 mg (77%), yellow oil. ^1^H NMR (300 MHz, CDCl_3_) *δ* 7.53 (d, 2H), 7.40 (t, 3H), 4.79 (s, 1H), 2.90–2.60 (m, 2H), 1.65–1.47 (m, 2H), 0.96 (t, *J* = 1.5 Hz, 3H) ppm.^[^ ^62^ ^]^

2‐(Isopropylamino)‐2‐phenylacetonitrile (**4ae**)

Yield: **Method B:** 77 mg (88%), yellow oil. ^1^H NMR (300 MHz, CDCl_3_) *δ* 7.56–7.49 (m, 2H), 7.45–7.36 (m, 3H), 4.78 (s, 1H), 3.23 (hept, *J* = 6.2 Hz, 1H), 1.16 (d, *J* = 6.2 Hz, 6H) ppm.^[^ ^14^ ^]^

2‐(Butylamino)‐2‐phenylacetonitrile (**4af**)

Yield: **Method B:** 61 mg (65%), yellow oil. ^1^H NMR (300 MHz, CDCl_3_) *δ* 7.52 (d, 2H), 7.37 (t, 3H), 4.78 (d, *J* = 1.6 Hz, 1H), 2.90–2.65 (m, 3H), 1.70–27 (m, 7H), 0.93 (t, 4H) ppm.^[^ ^63^ ^]^

2‐(*tert*‐Butylamino)‐2‐phenylacetonitrile (**4ag**)

Yield: **Method B:** 134 mg (71%), yellow oil. ^1^H NMR (300 MHz, CDCl_3_) *δ* 7.55–7.49 (m, 2H), 7.43–7.30 (m, 3H), 4.73 (s, 1H), 1.27 (s, 9H) ppm.^[^ ^64^ ^]^

4‐Methyl‐2‐(methylamino)pentanenitrile (**4fb)**

Yield: **Method B:** 61 mg (96%), yellow oil. ^1^H NMR (300 MHz, CDCl_3_) *δ* 3.54–3.40 (m, 1H), 2.52 (d, *J* = 1.5 Hz, 3H), 1.88 (dp, *J* = 13.3, 6.7 Hz, 1H), 1.71–1.49 (m, 2H), 0.93 (dt, *J* = 5.1, 2.4 Hz, 6H) ppm. ^13^C NMR (75 MHz, CDCl_3_) *δ* 120.19, 50.77, 42.12, 34.02, 24.91, 22.44, 21.95 ppm. HRMS (TOF‐MS): m/z [M + H]^+^ calculated for C_7_H_14_N_2_: 127.1229; found: 127.1235.

2‐(Ethylamino)‐4‐methylpentanenitrile (**4fc**)

Yield: **Method B:** 58 mg (82%), yellow oil. ^1^H NMR (300 MHz, CDCl_3_) *δ* 3.56 (dd, *J* = 8.3, 6.9 Hz, 1H), 3.00–2.84 (m, 1H), 2.70–2.56 (m, 1H), 1.96–1.82 (m, 1H), 1.66–1.56 (m, 2H), 1.12 (t, *J* = 7.1j Hz, 3H), 0.95 (d, *J* = 3.9 Hz, 3H), 0.93 (d, *J* = 3.9 Hz, 3H) ppm. ^13^C NMR (75 MHz, CDCl_3_) *δ* 120.50, 48.91, 42.43, 41.99, 24.93, 22.51, 21.88, 14.77 ppm. HRMS (TOF‐MS): m/z [M + H]^+^ calculated for C_8_H_16_N_2_: 141.1391; found: 141.1391.

4‐Methyl‐2‐(propylamino)pentanenitrile (**4fd)**

Yield: **Method B:** 59 mg (70%), yellow oil. ^1^H NMR (300 MHz, CDCl_3_) *δ* 3.72–3.67 (m, 0.5H), 3.55–3.47 (m, 0.5H), 2.85–2.76 (m, 0.5H), 2.68–2.51 (m, 1H), 1.95–1.78 (m, 1H), 1.60–1.55 (m, 1.5H), 1.49–1.43 (m, 1.5H), 1.41 (d, *J* = 1.6 Hz, 2H), 0.92 (d, *J* = 9.9 Hz, 6H) ppm. ^13^C NMR (75 MHz, CDCl_3_) *δ* 120.62, 49.48, 49.09, 42.46, 24.90, 22.88, 22.45, 21.93, 11.59 ppm. HRMS (TOF‐MS): m/z [M + H]^+^ calculated for C_9_H_18_N_2_: 155.1548; found: 155.1551.

2‐(Isopropylamino)‐4‐methylpentanenitrile (**4fe)**

Yield: **Method B:** 60 mg (77%), yellow oil. ^1^H NMR (300 MHz, CDCl_3_) *δ* 3.60—3.49 (m, 1H), 3.05 (dt, *J* = 12.4, 6.2 Hz, 1H), 1.97—1.77 (m, 1H), 1.64—1.56 (m, 2H), 1.41 (s, 1H), 1.10 (d, *J* = 6.4 Hz, 1H), 1.07 (d, *J* = 6.3 Hz, 3H), 0.99 (d, *J* = 6.1 Hz, 3H), 0.91 (d, *J* = 1.7 Hz, 3H), 0.89 (d, *J* = 1.7 Hz, 3H) ppm. ^13^C NMR (125 MHz, CDCl_3_) *δ* 120.70, 46.93, 46.52, 42.92, 29.32, 24.91, 23.77, 22.37, 21.98, 21.23 ppm. HRMS (TOF‐MS): m/z [M + H]^+^ calculated for C_9_H_18_N_2_: 155.1548; found: 155.1548.

2‐(Butylamino)‐4‐methylpentanenitrile (**4ff**)

Yield: **Method B:** 51 mg (60%) yellow oil. ^1^H NMR (500 MHz, CDCl_3_) *δ* 3.57 (t, *J* = 7.9 Hz, 1H), 2.87–2.79 (m, 0.5H), 2.68–2.59 (m, 1H), 1.93 (dh, *J* = 13.2, 6.6 Hz, 1H), 1.72–1.58 (m, 2H), 1.56–1.45 (m, 2H), 1.41 (dt, *J* = 15.0, 7.4 Hz, 2H), 0.98 (dd, *J* = 6.4, 5.0 Hz, 6H), 0.94 (d, *J* = 7.3 Hz, 3H) ppm. ^13^C NMR (125 MHz, CDCl_3_) *δ* 120.65, 49.18, 47.40, 42.54, 31.84, 24.96, 22.48, 21.98, 20.30, 13.87 ppm. HRMS (TOF‐MS): m/z [M + H]^+^ calculated for C_10_H_20_N_2_: 169.1704; found: 169.1708.

2‐(*tert*‐Butylamino)‐4‐methylpentanenitrile (**4fg**)

Yield: **Method B:** 57 mg (68%) yellow oil. ^1^H NMR (300 MHz, CDCl_3_) *δ* 3.49 (d, *J* = 1.6 Hz, 1H), 1.93–1.81 (m, 2H), 1.66–1.57 (m, 1H), 1.15 (dd, *J* = 2.0, 1.0 Hz, 9H), 0.91 (td, *J* = 4.7, 2.5 Hz, 6H) ppm. ^13^C NMR (75 MHz, CDCl_3_) *δ* 123.33, 51.31, 44.86, 42.12, 29.33, 24.55, 22.24, 22.09 ppm. HRMS (TOF‐MS): m/z [M + H]^+^ calculated for C_10_H_20_N_2_: 169.1704; found: 169.1704.

##### AtHNL and HbHNL Enzyme Expression

Expression plasmids for AtHNL and HbHNL were a kind gift from Prof. Romas Kazlauskas.^[^ ^49^ ^]^ Plasmids were transformed into competent *E. coli* BL21(DE3) cells. Cells were grown in LB culture medium at 37 °C. After reaching OD_600_ = 0.8–1.0, cells were induced by the addition of IPTG (isopropyl ß‐D‐1‐thiogalactopyranoside). Protein expression was done overnight at 18 °C. Cells were harvested by centrifugation, then resuspended in HEPES buffer (50 mM HEPES, 150 mM NaCl, pH 7.5). Cells were disrupted by ultrasonic homogenizer (Bandelin, Sonoplus HD 4000). The lysate was separated from the cell debris by centrifugation. In the next step, the cell lysate was purified on a Ni‐resin gravity column. HNLs were eluted by 250 mM imidazole (in 50 mM HEPES, 150 mM NaCl, pH 7.5 buffer). Imidazole was removed via Cytiva PD‐10 desalting column. The desalted proteins were concentrated through Amicon Ultra Centrifugal Filter (MWCO 10 kDa). The concentration of the protein solution was measured by NanoDrop (by Thermo Fischer Scientific). Purity of the enzymes was assessed with SDS‐PAGE. The concentrated final protein solutions were stored in a freezer at −80 °C after the addition of 10 v*/v*% glycerol until use.

##### Computational Details

For the docking calculations X‐ray structure of HbHNL in complex with mandelonitrile (PDB id: 1YB6) protein was used, which was prepared using standard settings of Protein Preparation Wizard (Maestro version: 11.9.011, Protein Preparation Wizard, Schrödinger, LLC, New York, NY, 2016).^[^ ^65^ ^]^ The ligands were prepared with Ligprep (Schrödinger Suite, 2019−1; Schrödinger, LLC: New York, NY, 2019. Maestro version: 11.9.011) using standard settings. The docking calculations were performed with Induced Fit Docking of Schrödinger (Induced Fit Docking protocol; Glide, Schrödinger, LLC, New York, NY, 2016; Prime, Schrödinger, LLC, New York, NY, 2019),^[^ ^66^ ^]^ where 20 poses were generated for each ligand, and the top 20 protein structures were selected for redocking within 30 kcal mol^−1^ of the best structure, using standard precision method.

## Conflict of Interest

The authors declare no conflict of interest.

## Supporting information

Supplementary Material

## Data Availability

The data that support the findings of this study are available from the corresponding author upon reasonable request.
