# Supplementary material for: Buffer‐Mediated Catalyst‐Free Strecker Reaction Toward Enzymatic Implementation
Source: ChemistryOpen. 2025 Sep 21;14(12):e202500389. doi: 10.1002/open.202500389 (PMC12680554; doi:10.1002/open.202500389)

Supplementary Material

Buffer-mediated catalyst-free Strecker reaction towards enzymatic implementation

Péter Magyar,[1,2,3] Szilárd Újvári,[1,2], Zsófia Molnár,[2] Zoltán Orgován,[1,3] Diána Balogh-Weiser,[2,4] Blanka Nagy,[5] Diana Maria Scrob[5], László Poppe,[2,5] and Péter Ábrányi-Balogh[1,2,3,]*

[1]Medicinal Chemistry Research Group, HUN-REN Research Centre for Natural Sciences, Magyar tudósok körútja 2. 1117 Budapest, Hungary.

[2]Department of Organic Chemistry and Technology, Faculty of Chemical Technology and Biotechnology, Budapest University of Technology and Economics, Műegyetem rkp 3. 1111 Budapest, Hungary

[3]National Drug Research and Development Laboratory, HUN-REN Research Centre for Natural Sciences, Magyar tudósok körútja 2. 1117 Budapest, Hungary

[4]Department of Physical Chemistry and Materials Science, Budapest University of Technology and Economics, Műegyetem rkp. 3, 1111 Budapest, Hungary

[5]Enzymology and Applied Biocatalysis Research Center, Faculty of Chemistry and Chemical Engineering, Babeș-Bolyai University, Arany János Street 11, 400028, Cluj-Napoca, Romania

*for correspondence: abranyi-balogh.peter@ttk.hu

Table S1. Control reactions in MTBE:PBS buffer (500 mM) 4:1 of **1a** and **2c**. Reaction time 4 h, enzyme concentration 2 µM.

pH	Enzyme	Isolated yield (**4ac**, %)
6	-	21
6	*At*HNL	81
6	*Hb*HNL	80
7	-	15
7	*At*HNL	60
7	*Hb*HNL	56
8	-	10
8	*At*HNL	43
8	*Hb*HNL	40

2-(Benzylamino)-2-phenylacetonitrile (**4aa**)

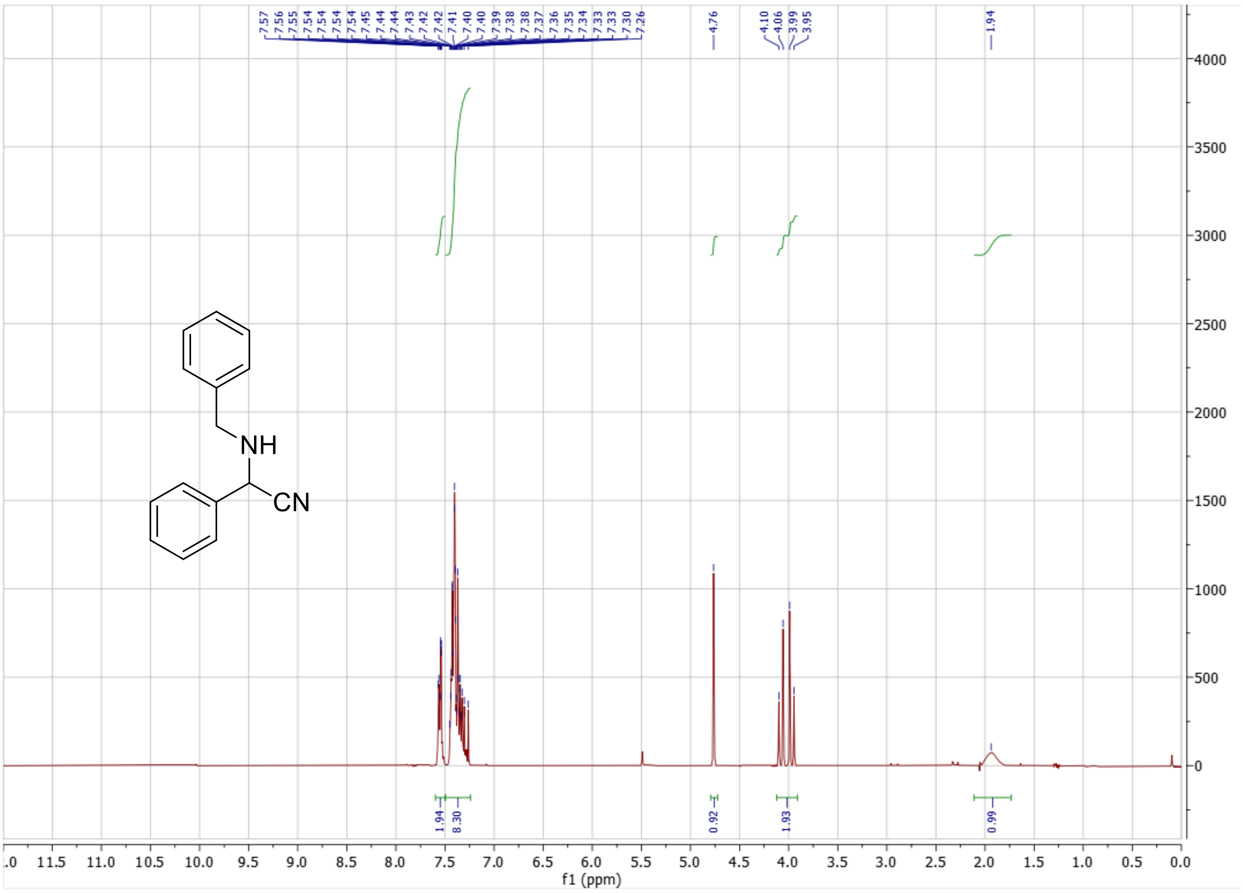

2-(Methylamino)-2-phenylacetonitrile (**4ab**)

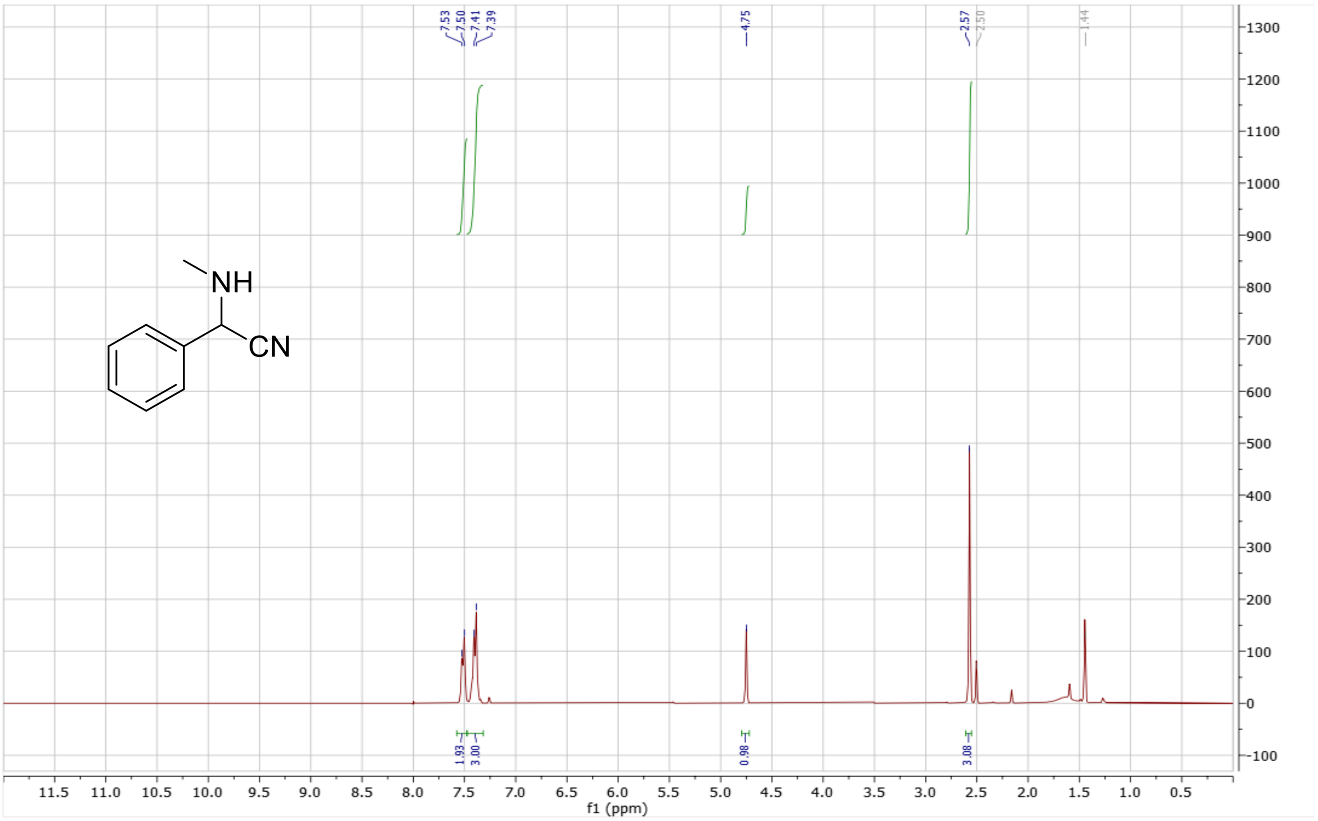

2-(Ethylamino)-2-phenylacetonitrile (**4ac**)

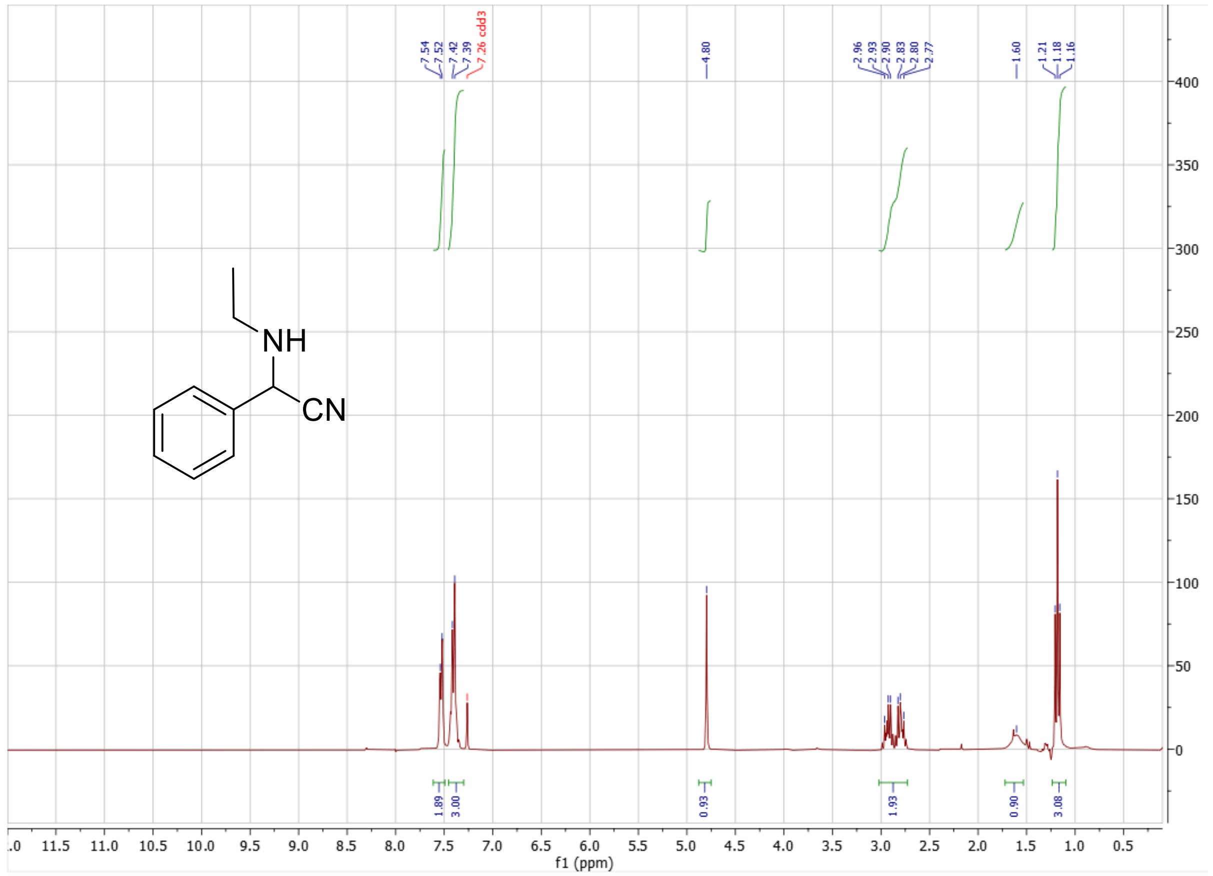

2-Phenyl-2-(propylamino)acetonitrile (**4ad**)

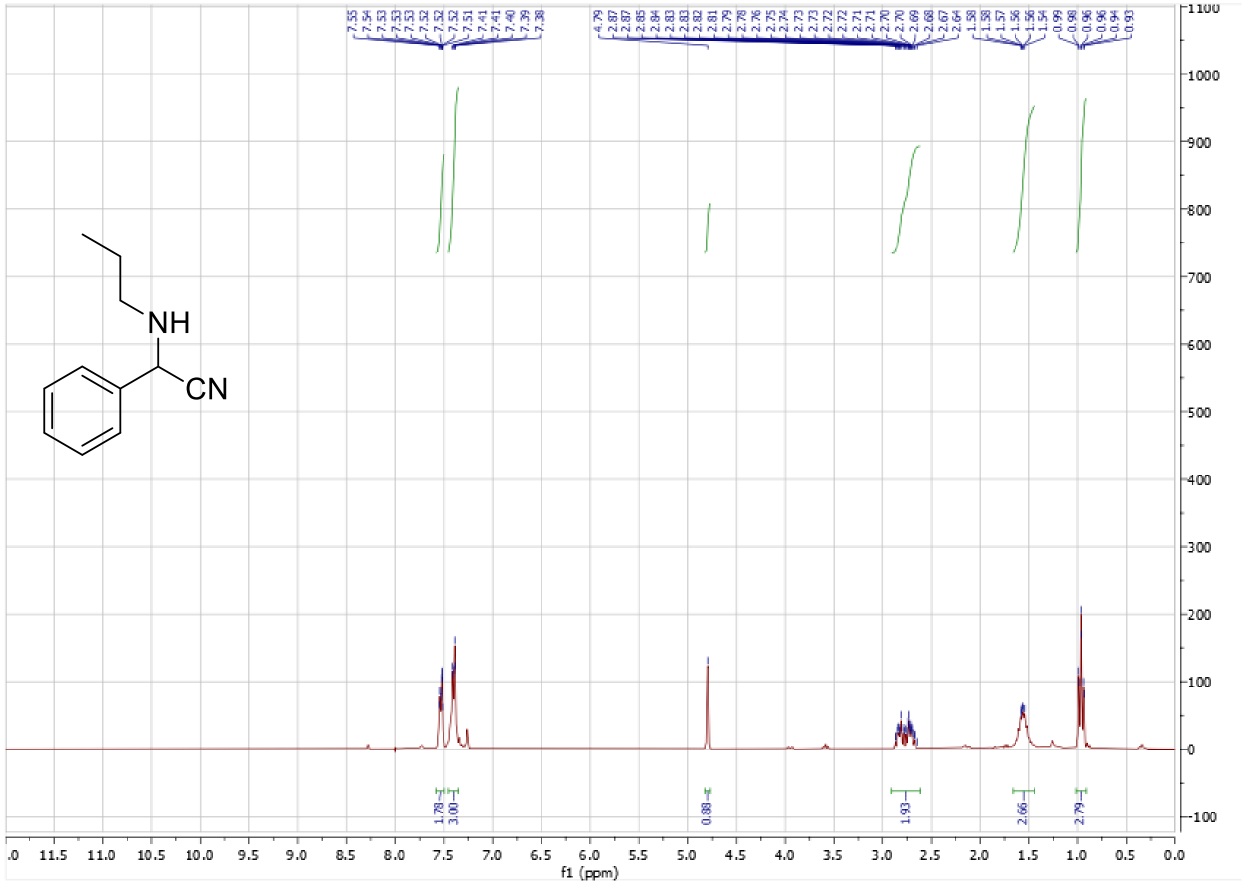

2-(Isopropylamino)-2-phenylacetonitrile (**4ae**)

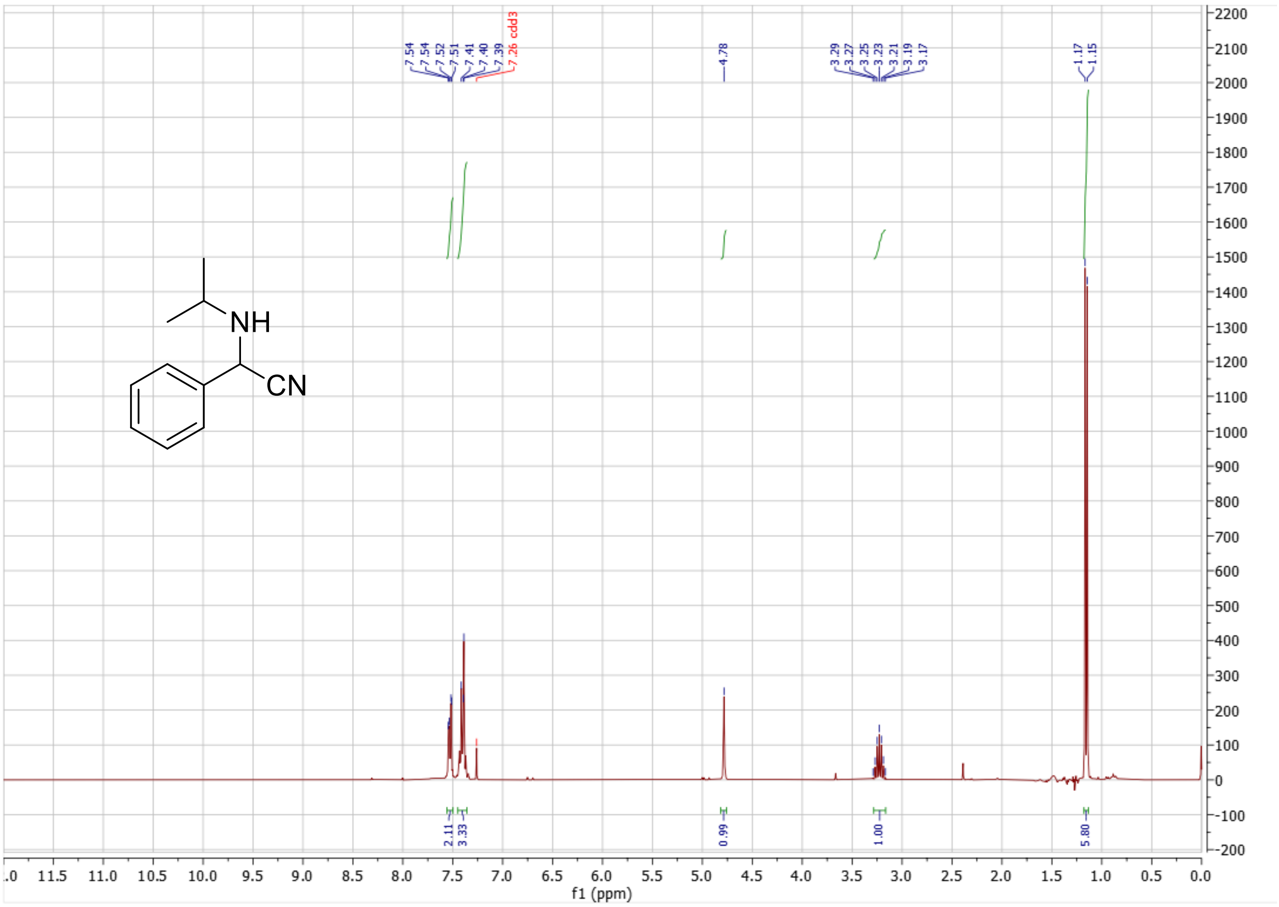

2-(Butylamino)-2-phenylacetonitrile (**4af**)

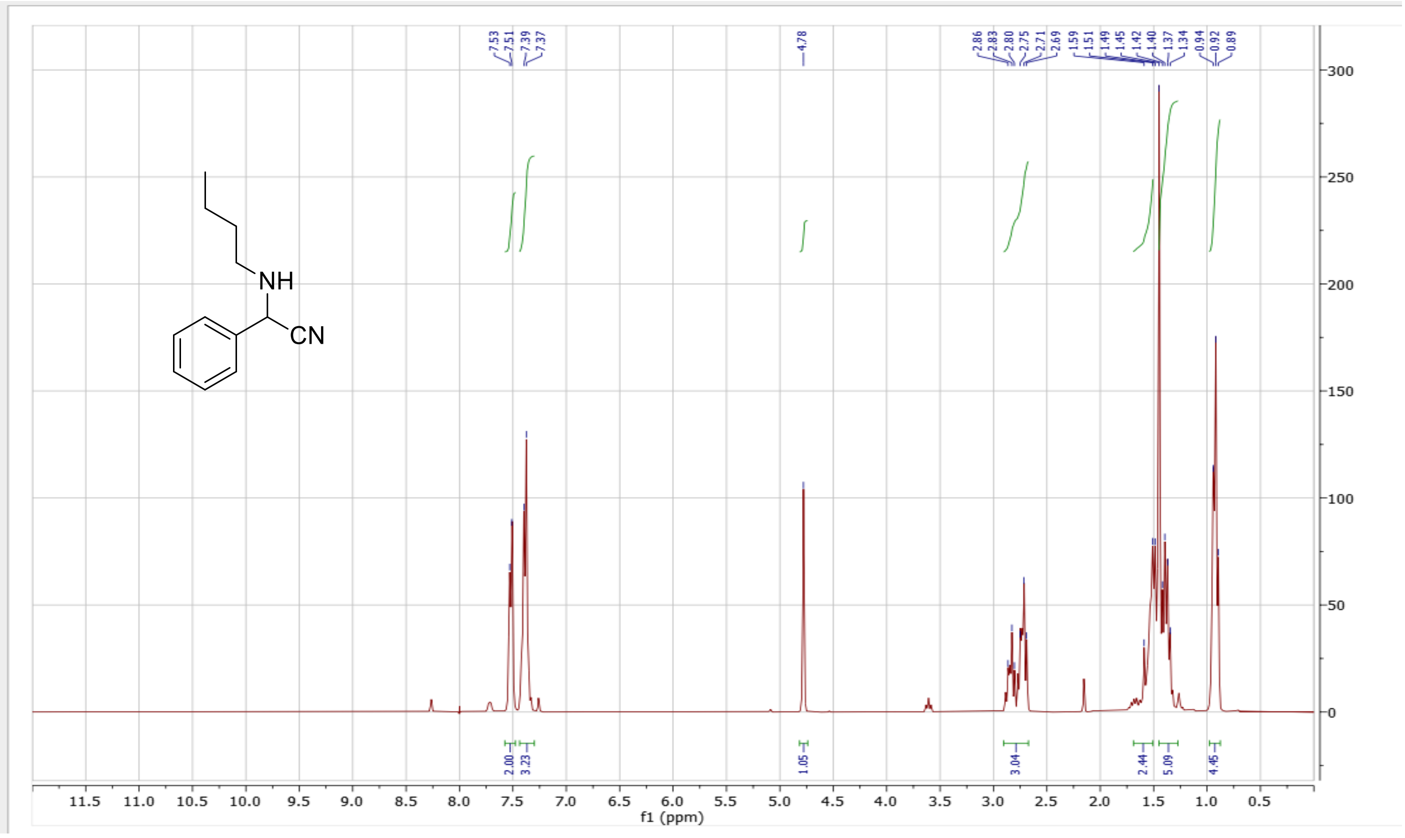

2-(*tert*-butylamino)-2-phenylacetonitrile (**4ag**)

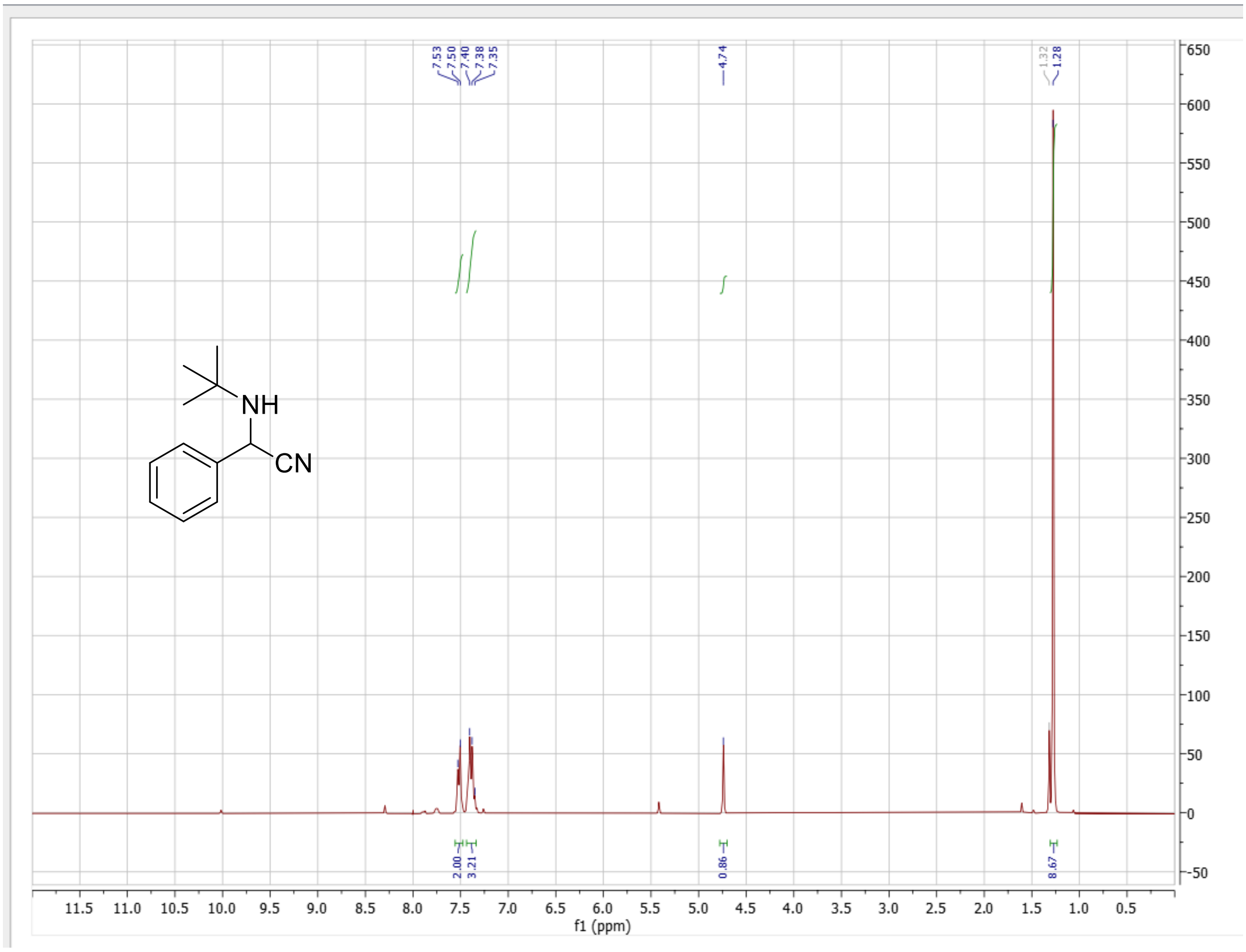

2-(Benzylamino)acetonitrile (**4ba**)

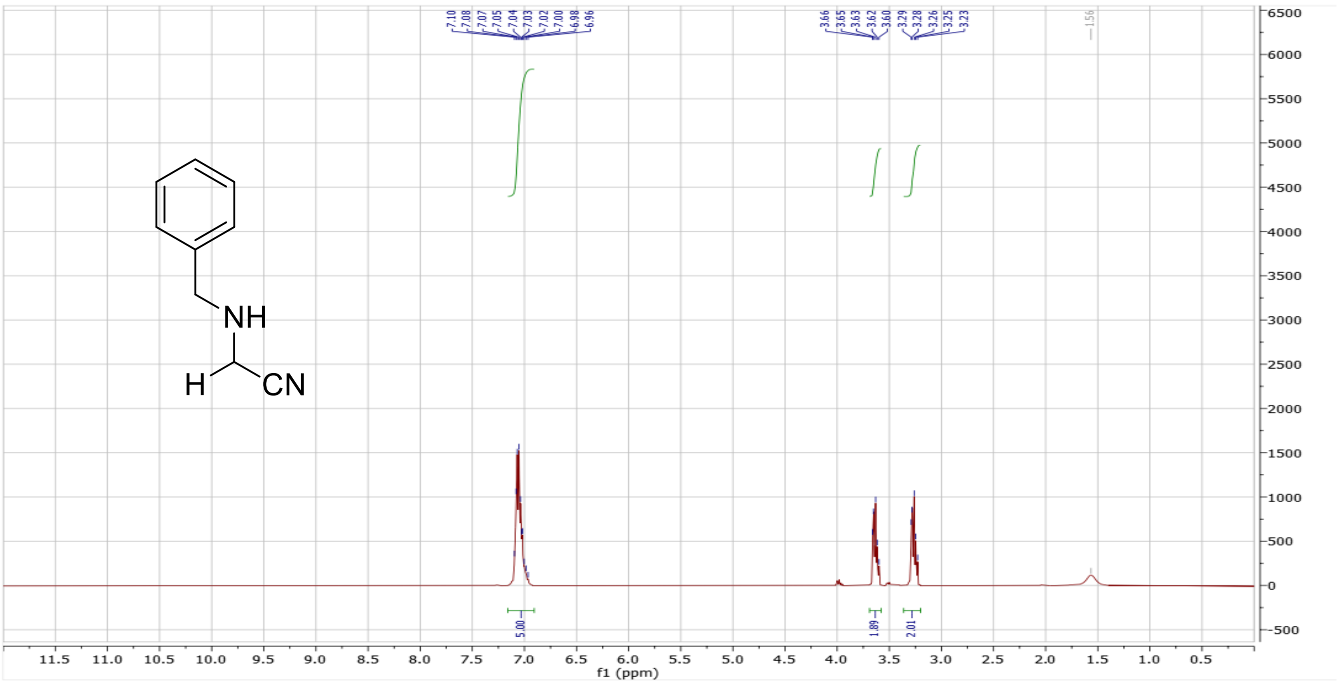

2-(Benzylamino)propanenitrile (**4ca**)

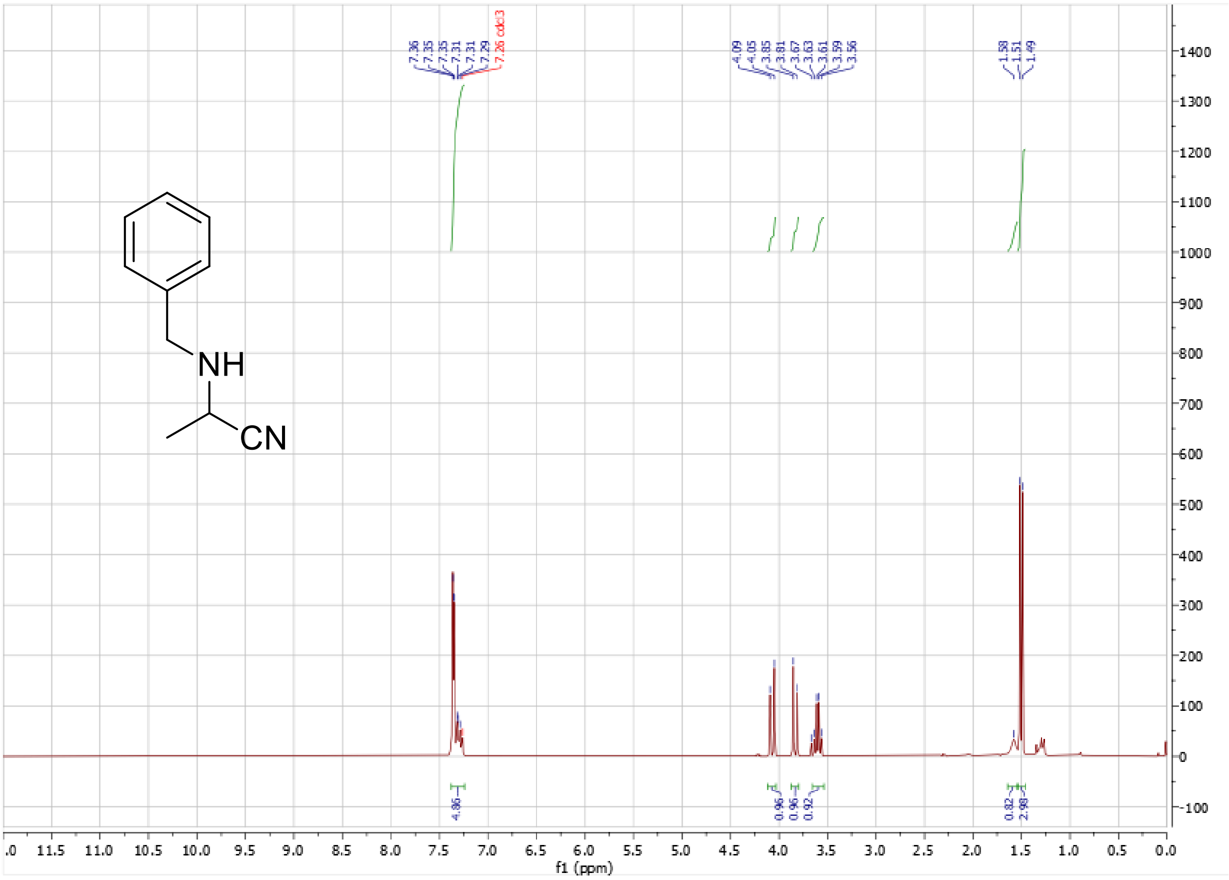

2-(Benzylamino)-2-methylpropanenitrile (**4da**)

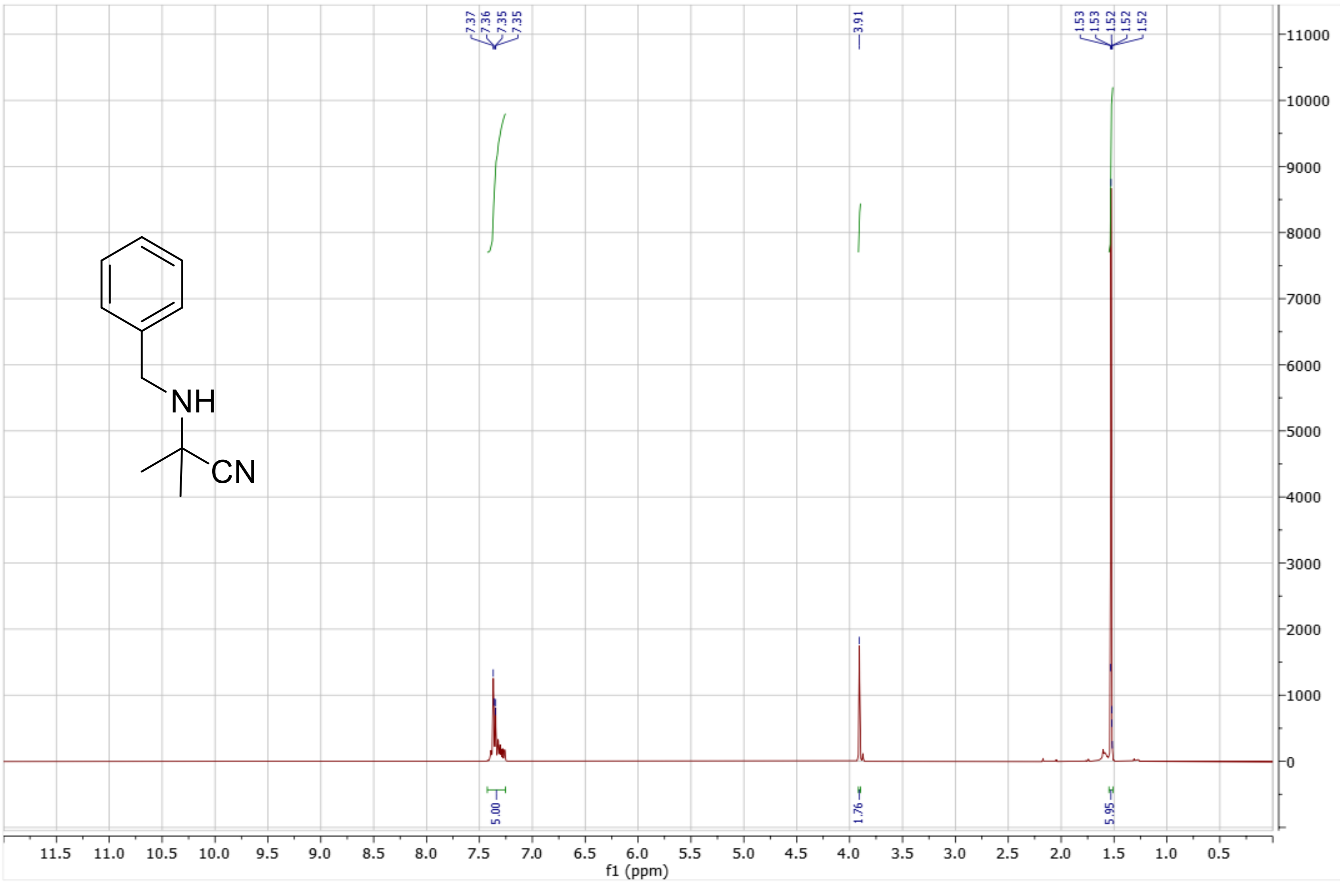

2-(Benzylamino)-3-methylbutanenitrile (**4ea**)

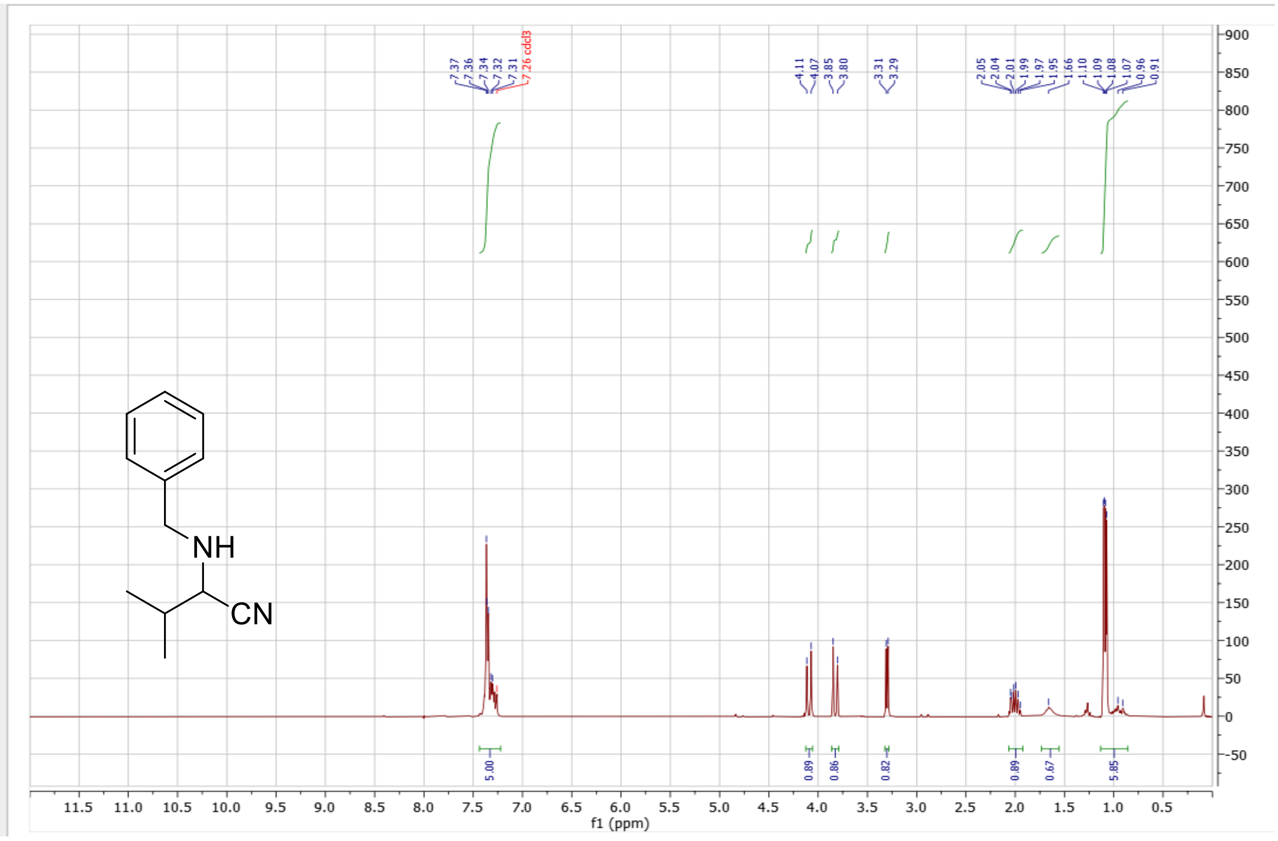

2-(Benzylamino)-4-methylpentanenitrile (**4fa**)

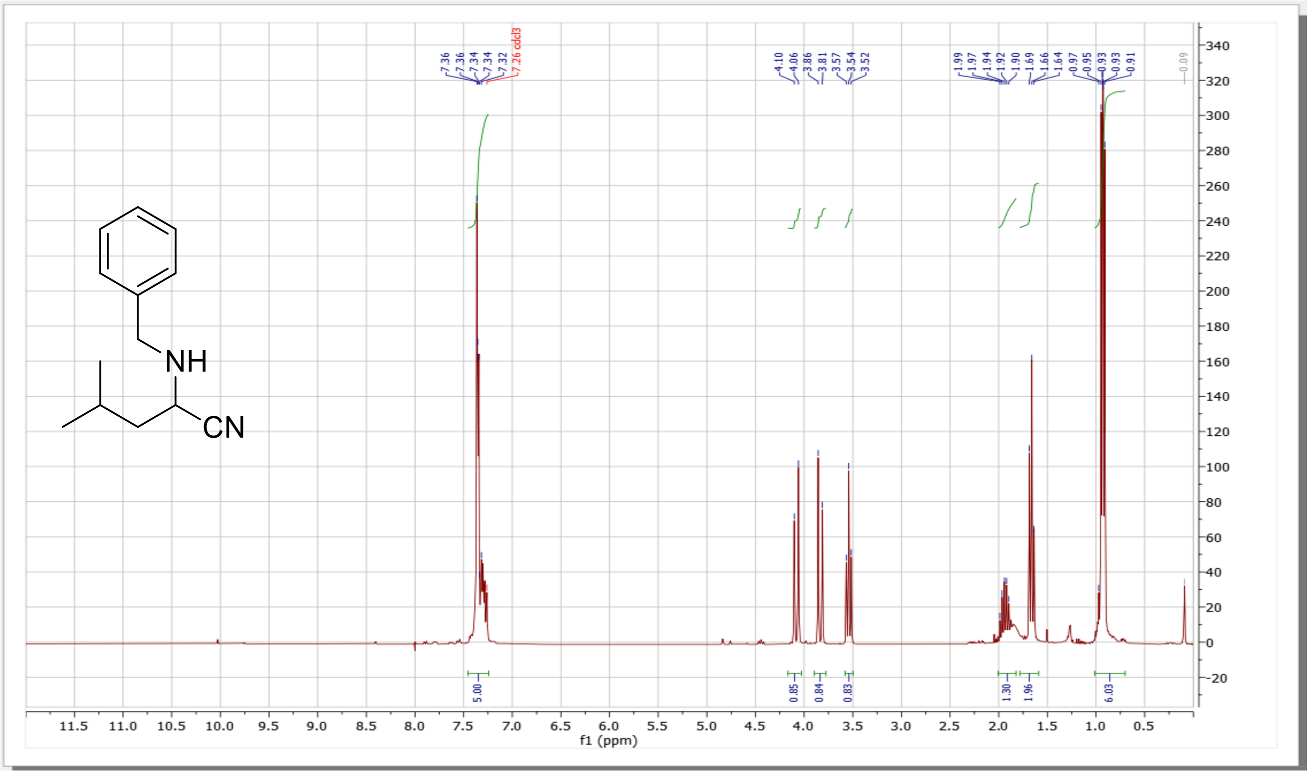

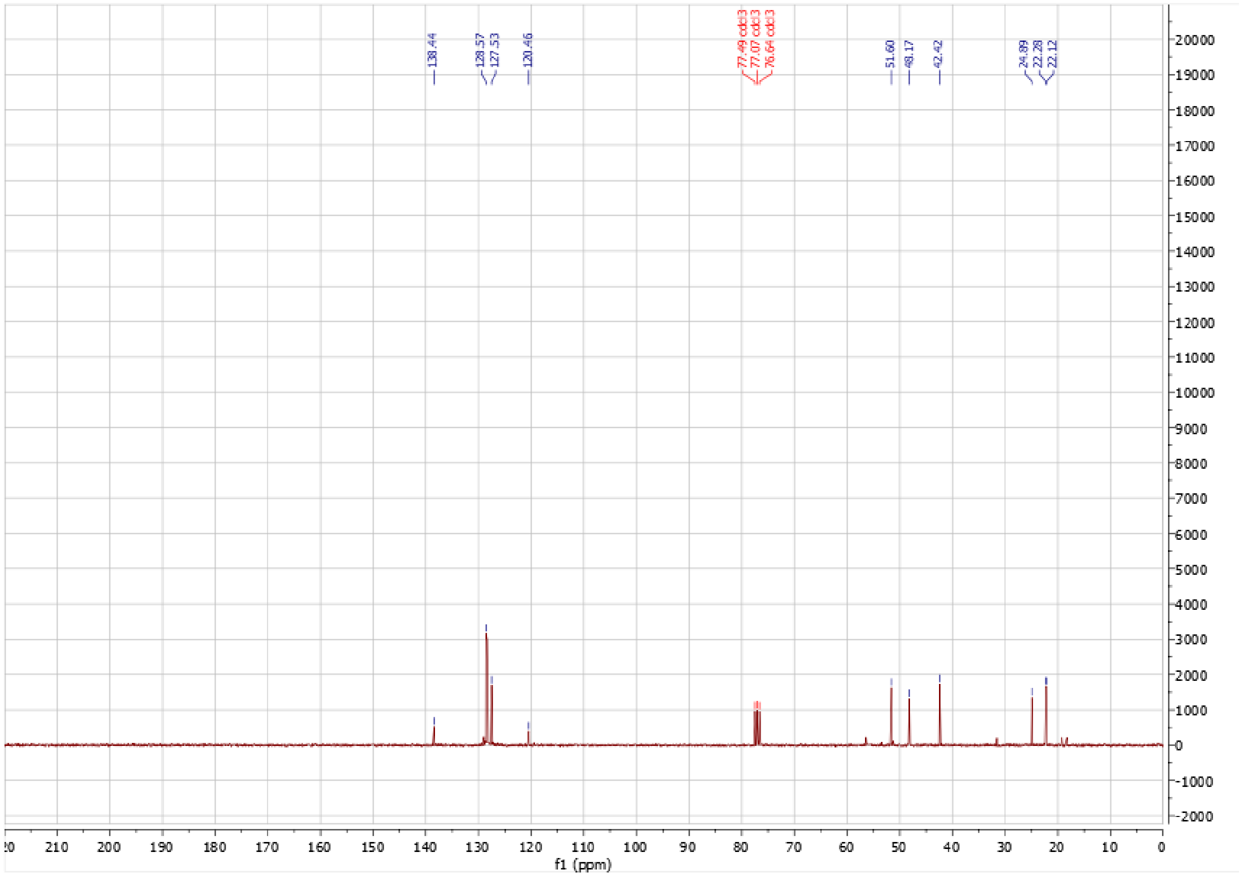

4-Methyl-2-(methylamino)pentanenitrile (**4fb**)

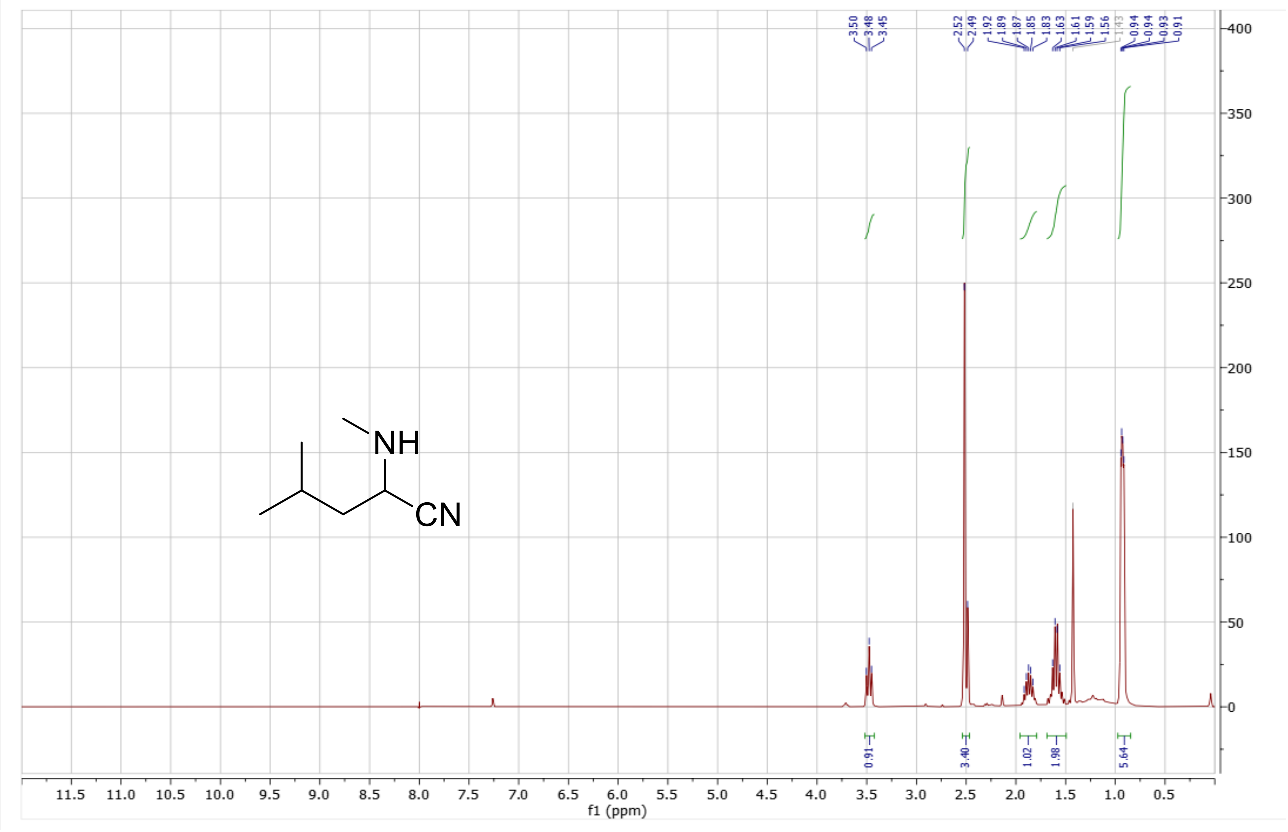

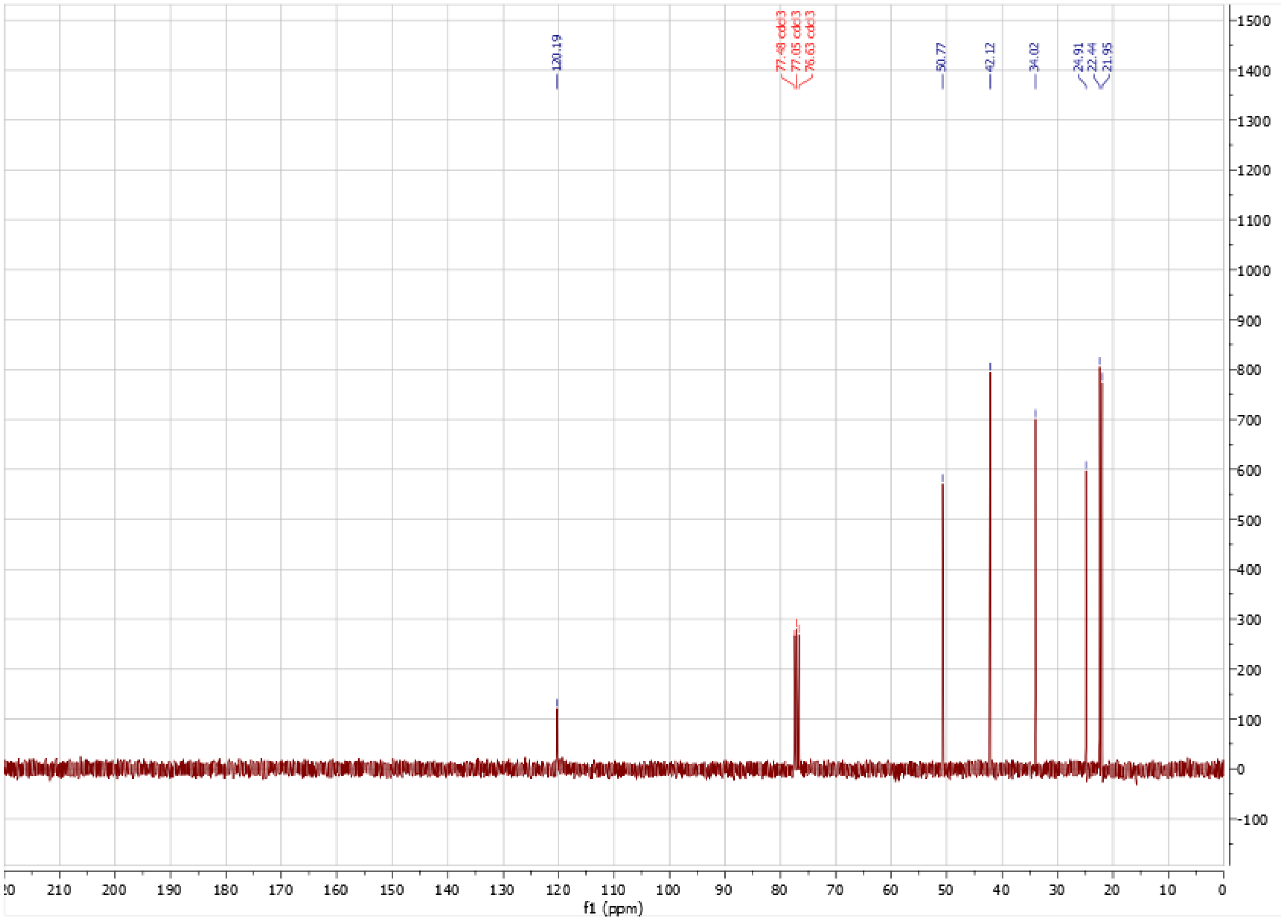

120.19
77.48 cdcl3
77.05 cdcl3
76.63 cdcl3
50.77
42.12
34.02
24.91
22.44
21.95
f1 (ppm)

2-(Ethylamino)-4-methylpentanenitrile (**4fc**)

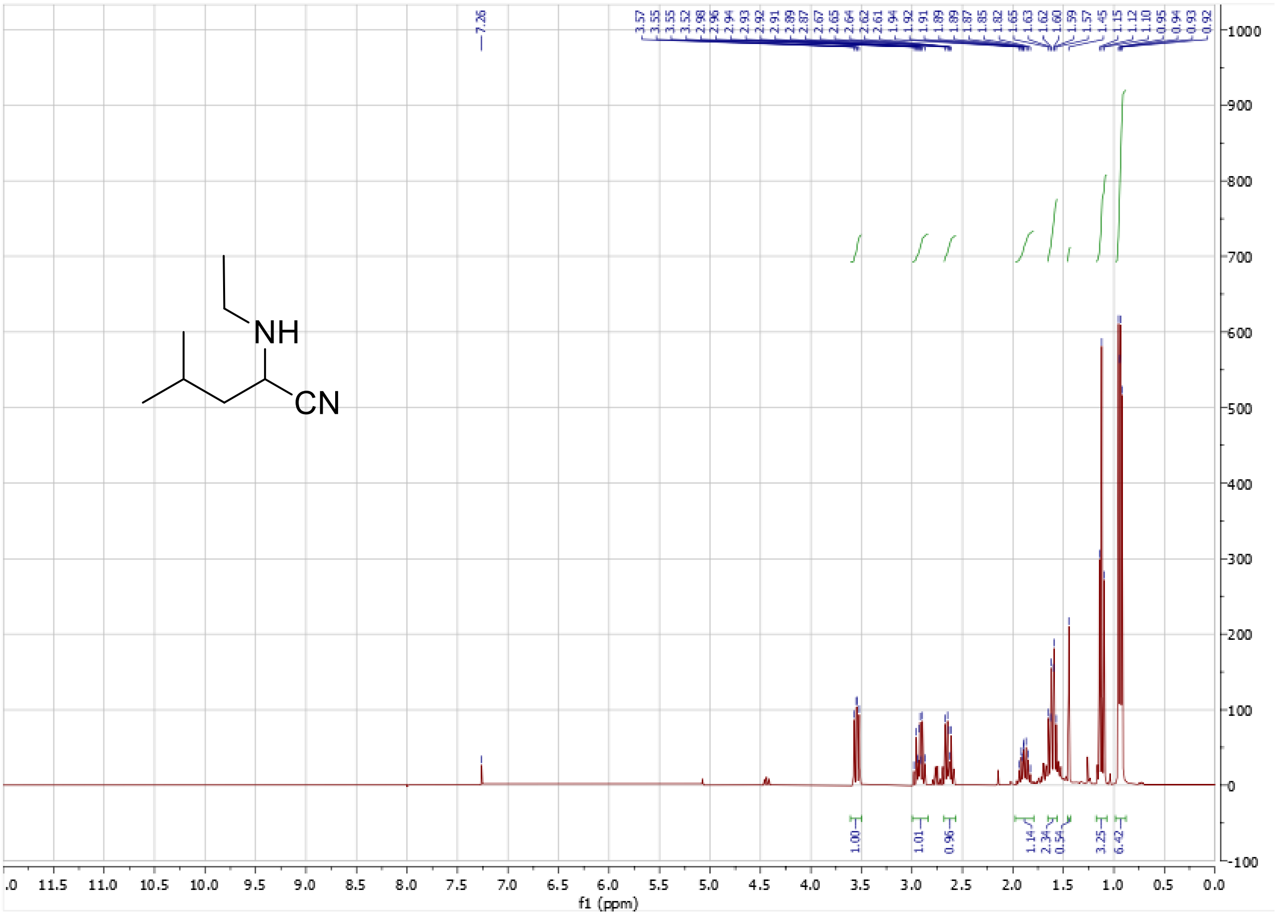

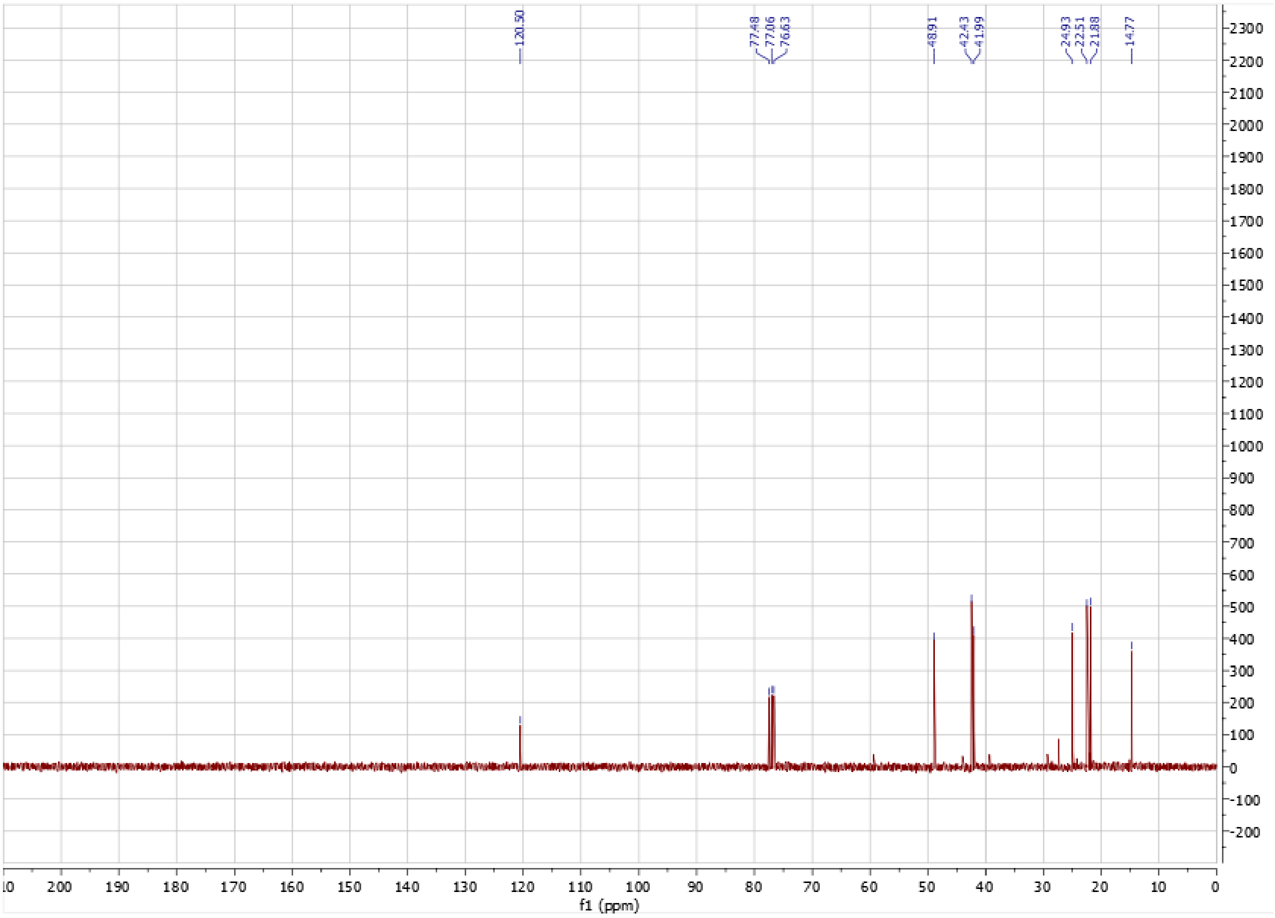

4-Methyl-2-(propylamino)pentanenitrile (**4fd**)

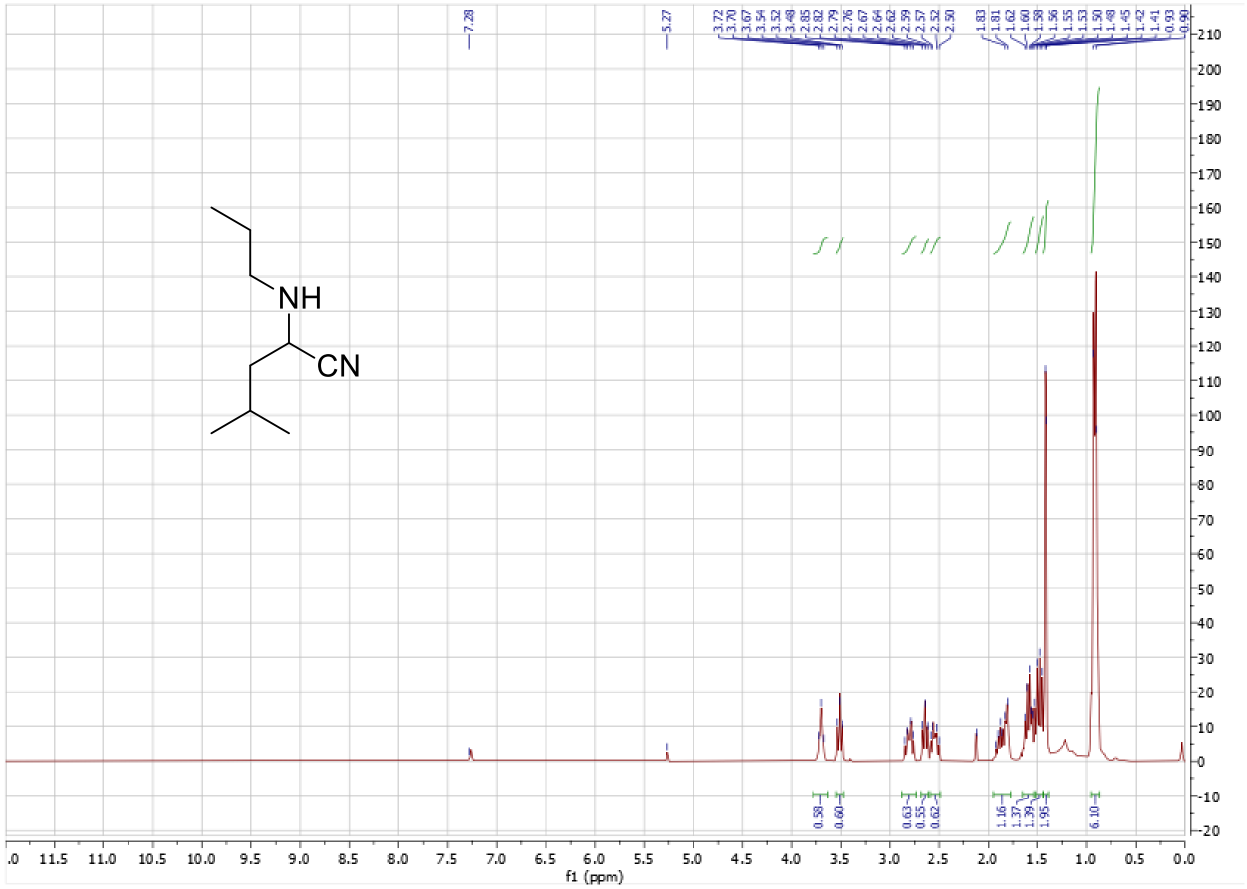

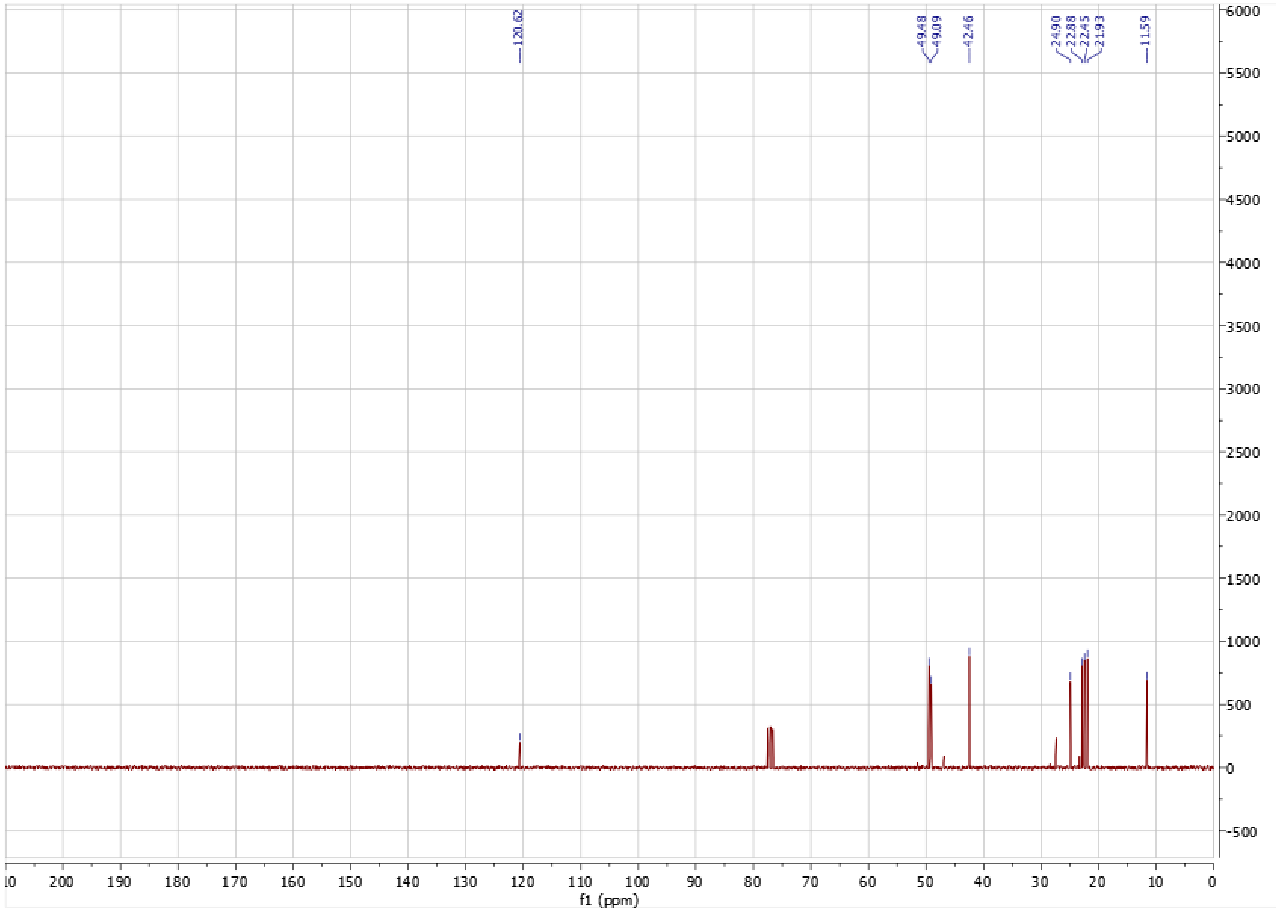

2-(Isopropylamino)-4-methylpentanenitrile (**4fe**)

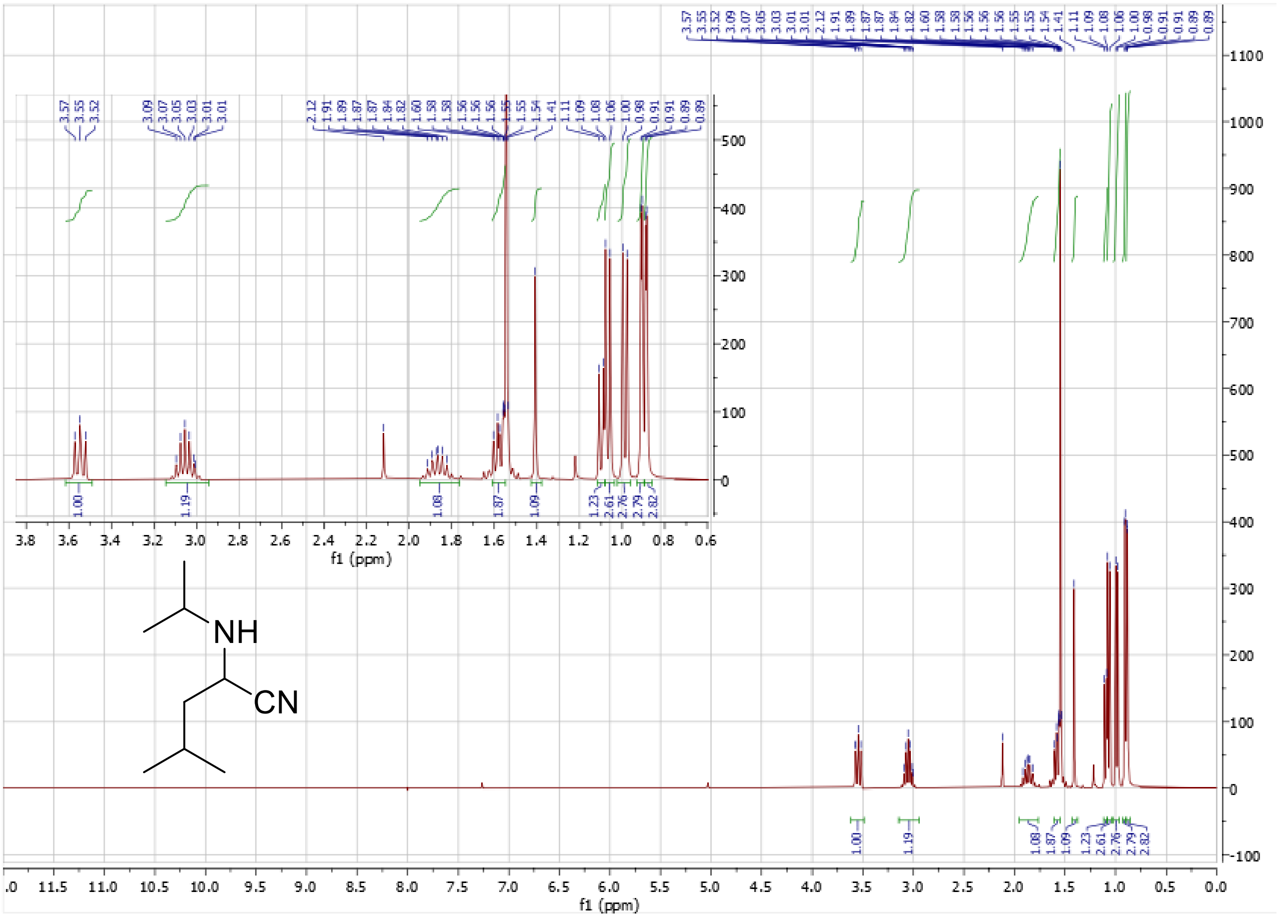

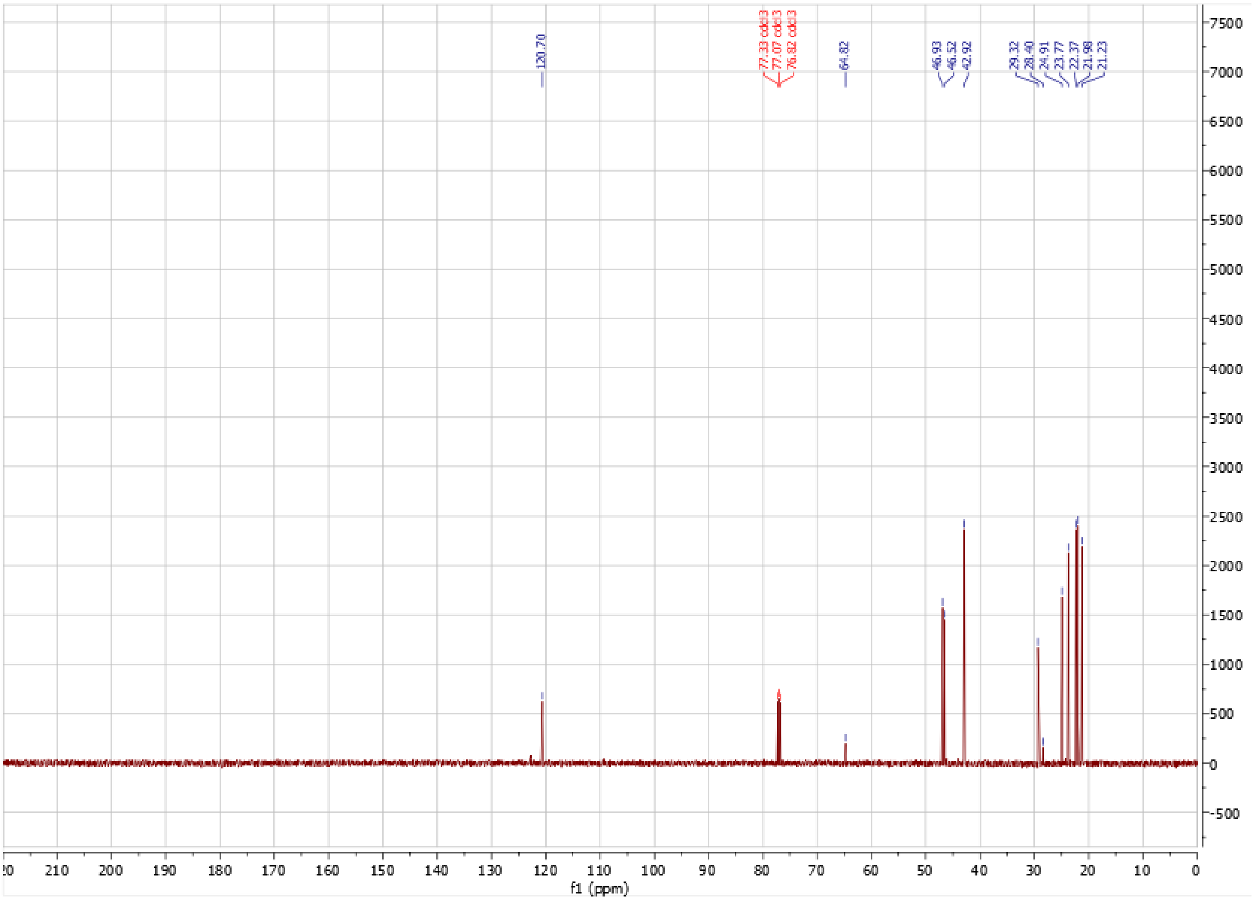

2-(Butylamino)-4-methylpentanenitrile (**4ff**)

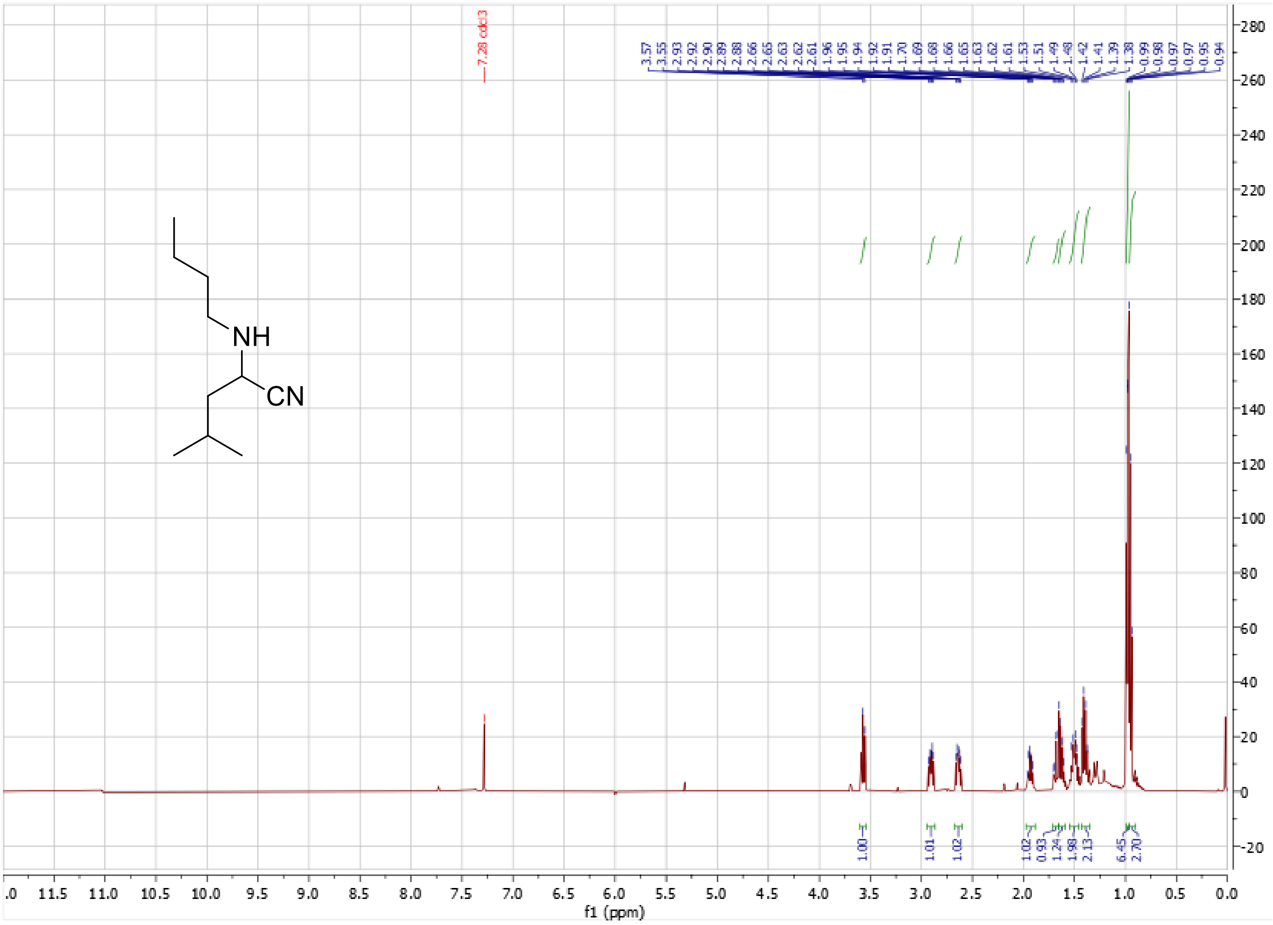

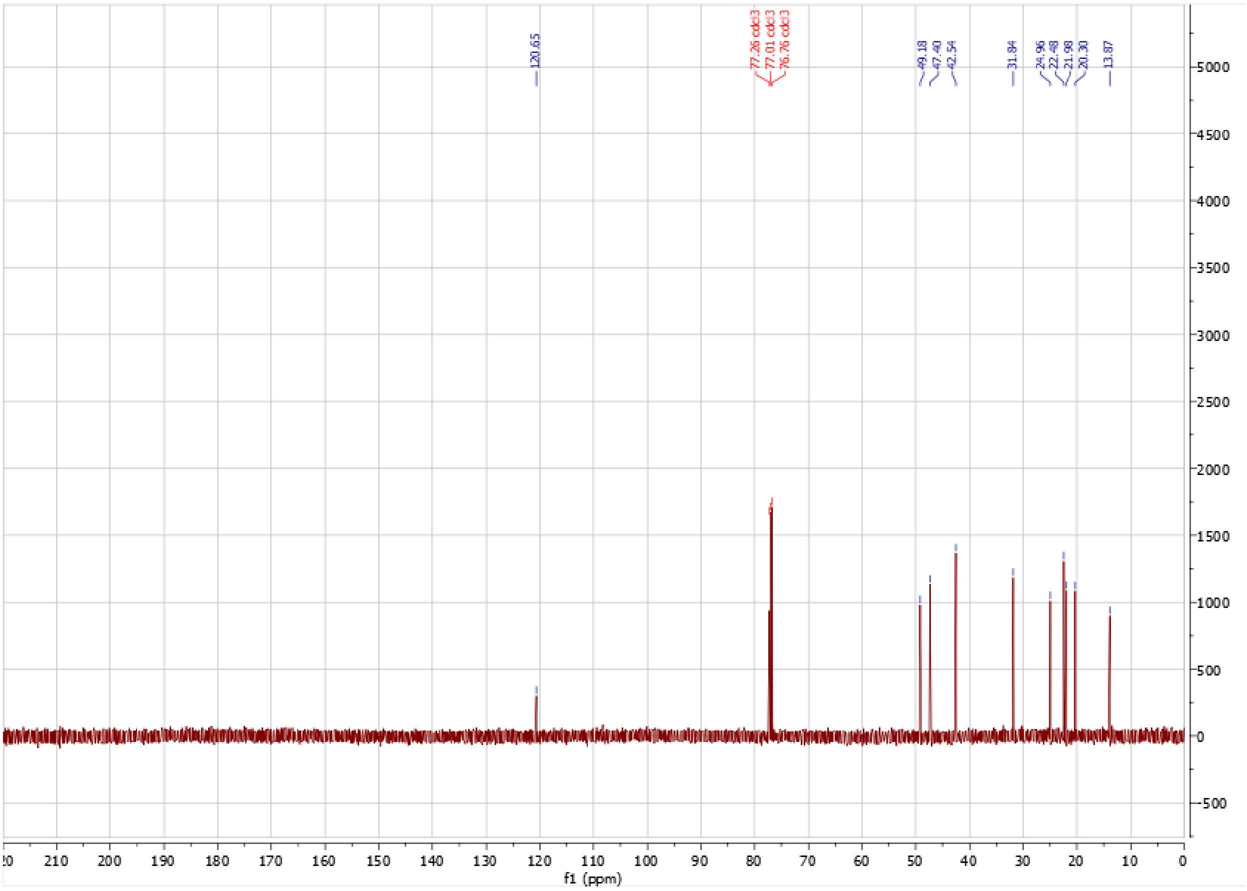

2-(*tert*-butylamino)-4-methylpentanenitrile (**4fg**)

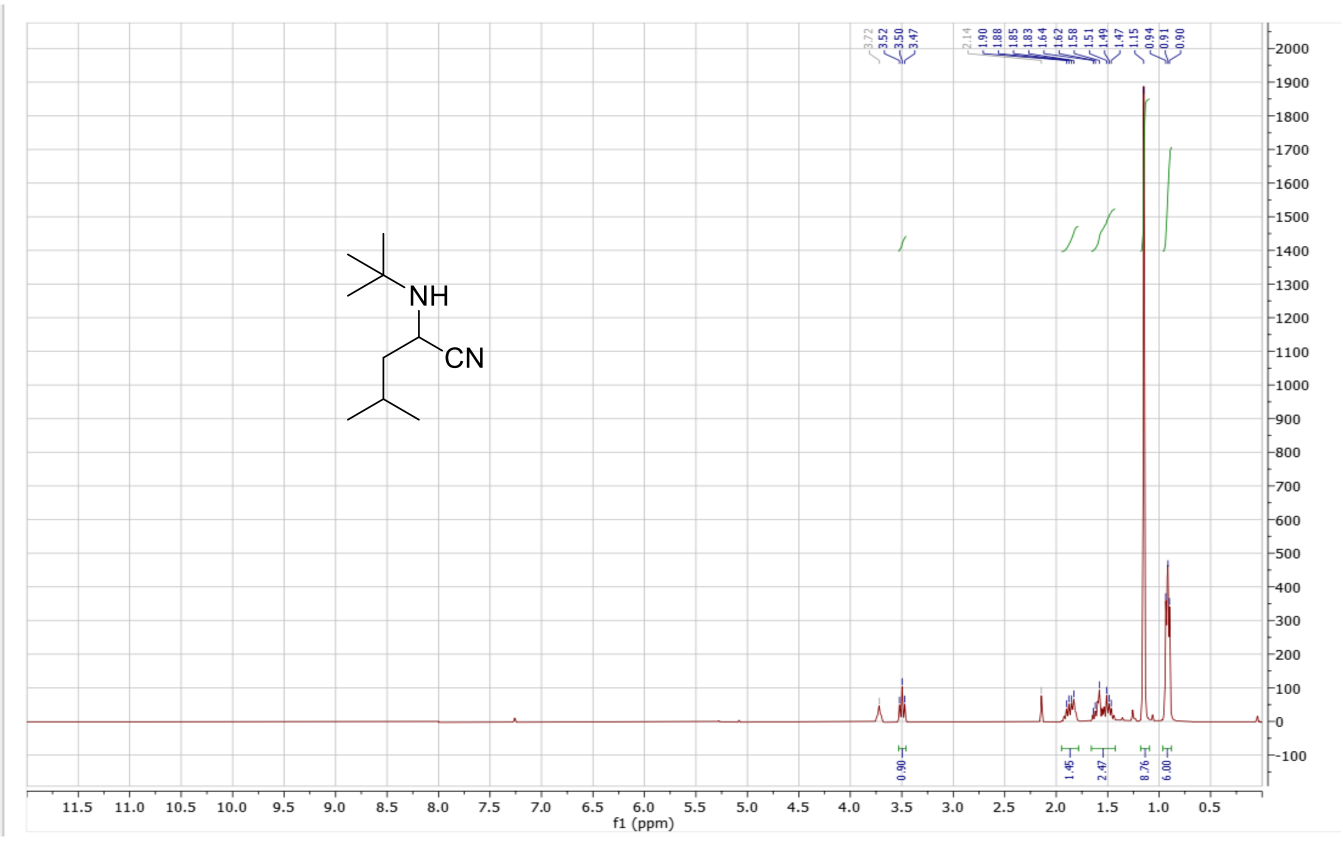

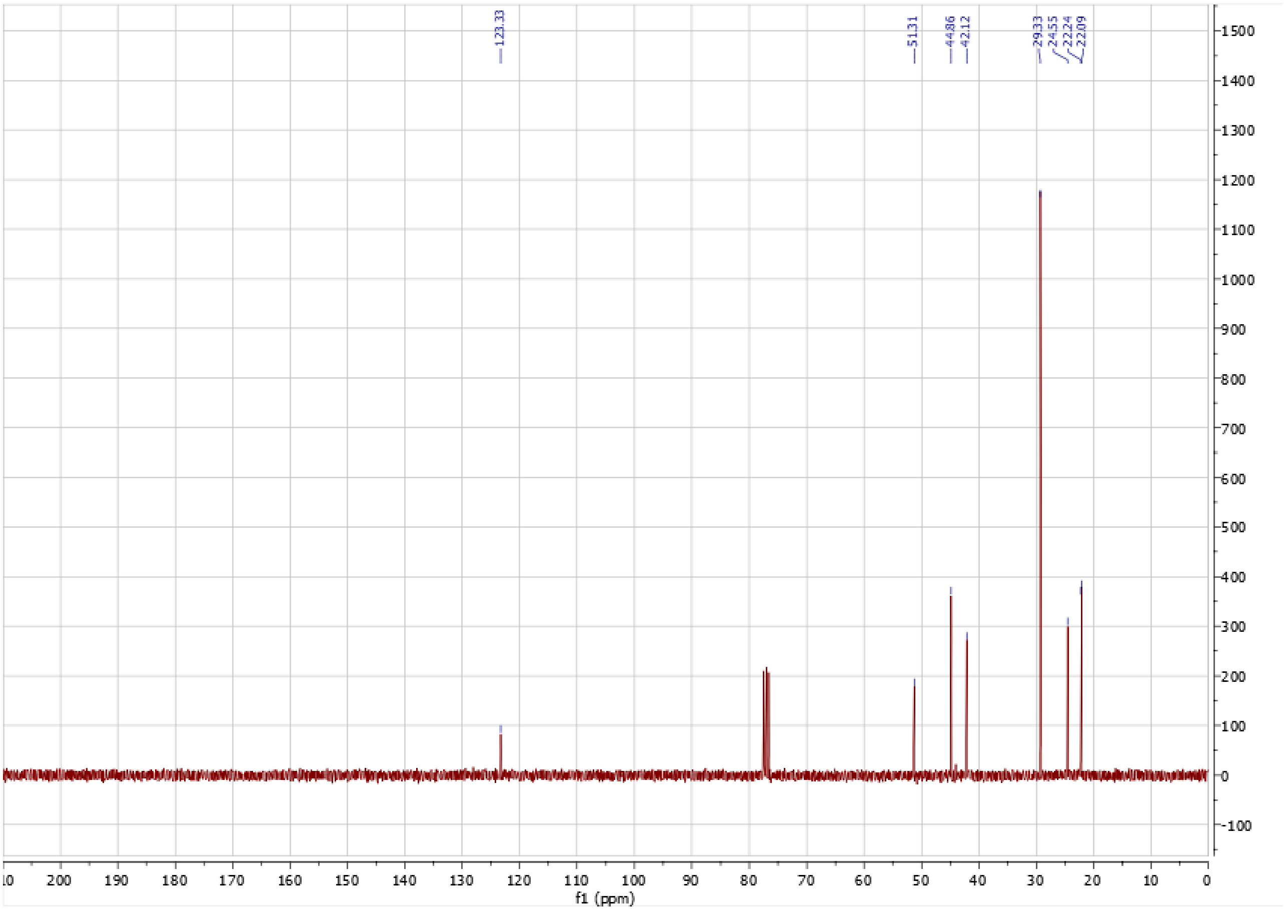

2-(Benzylamino)-2,2-diphenylacetonitrile (**4ga**)

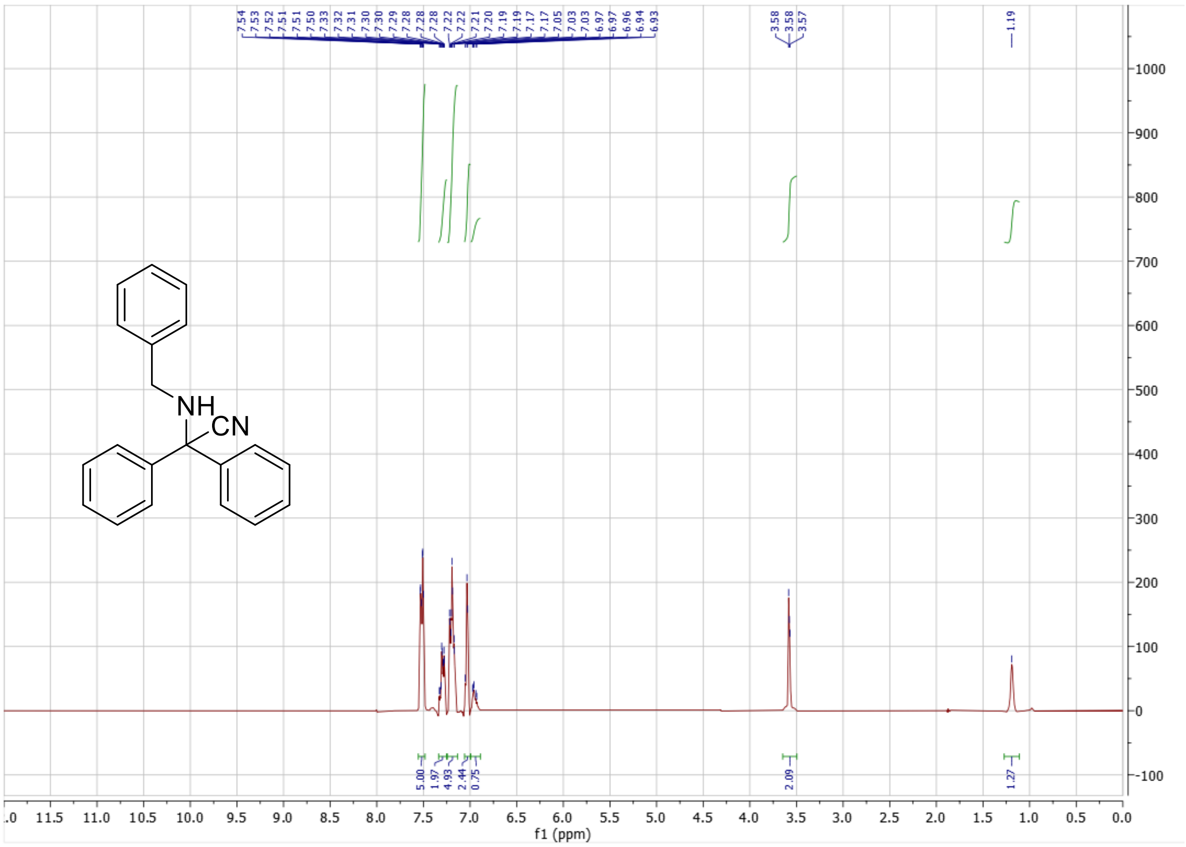

2-(Benzylamino)-4-(methylthio)butanenitrile (**4ha)**

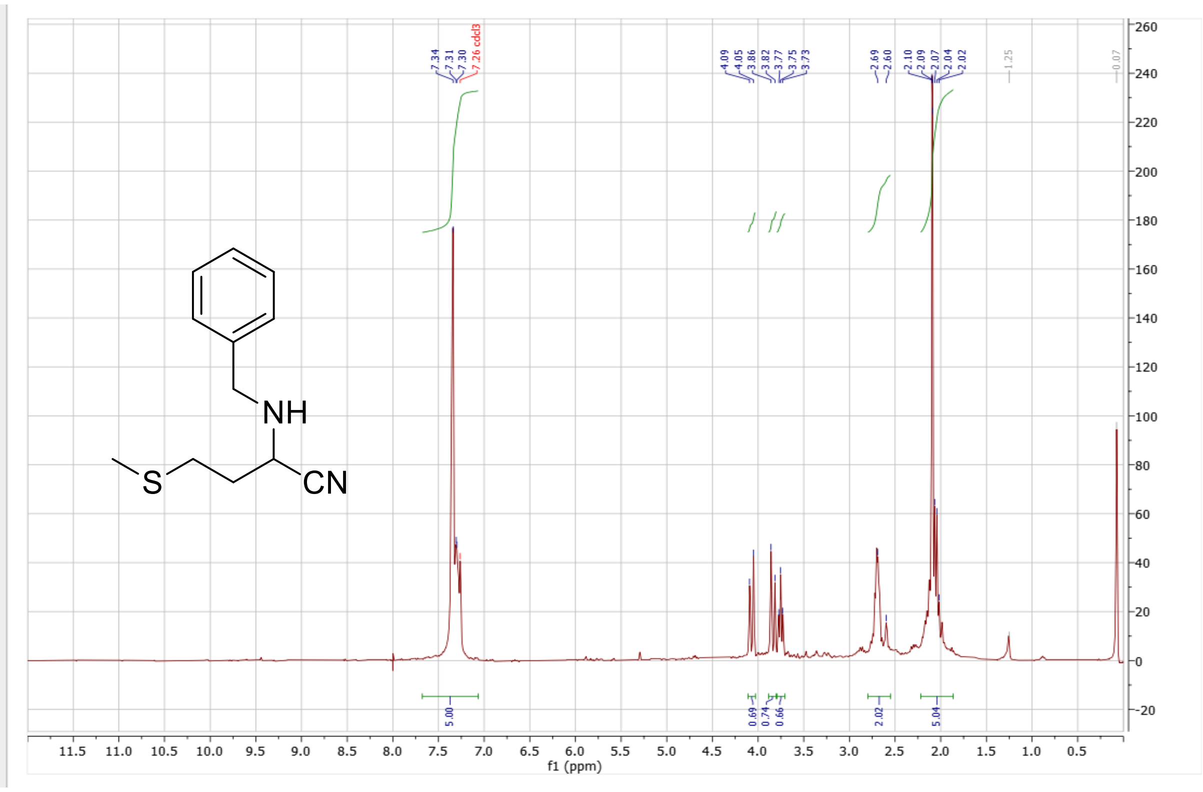

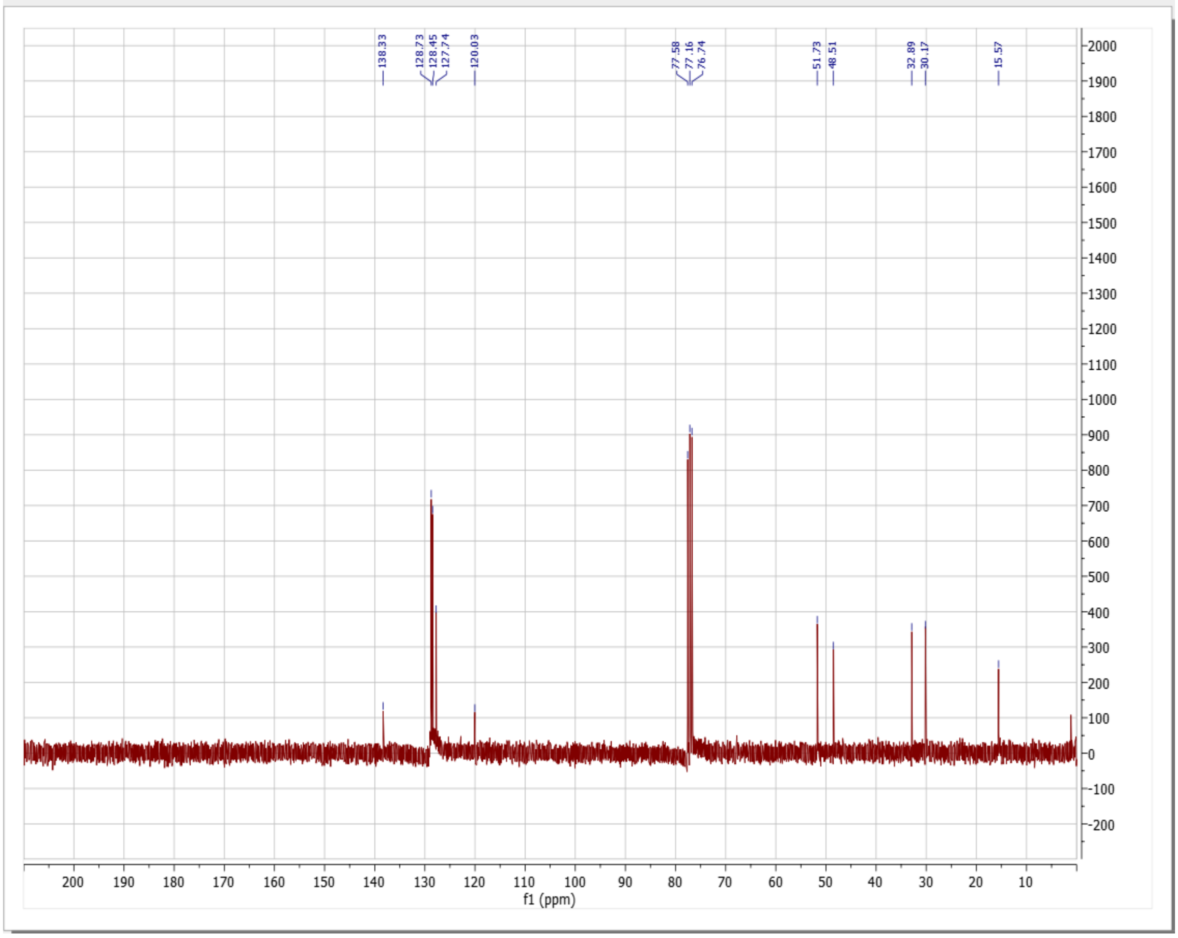

2-(Benzylamino)-2-(2-chlorophenyl)acetonitrile (**4ia**)

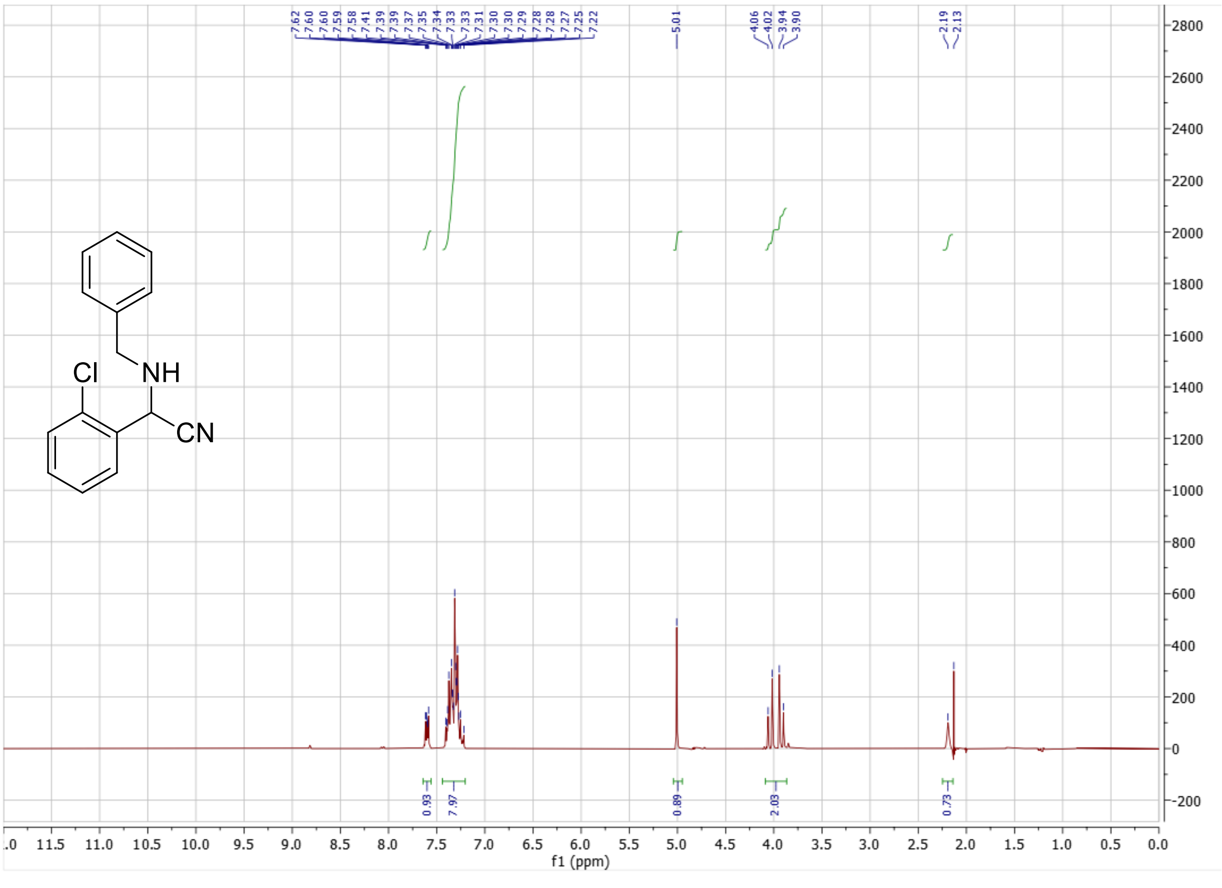

2-(Benzylamino)-2-(3-chlorophenyl)acetonitrile (**4ja**)

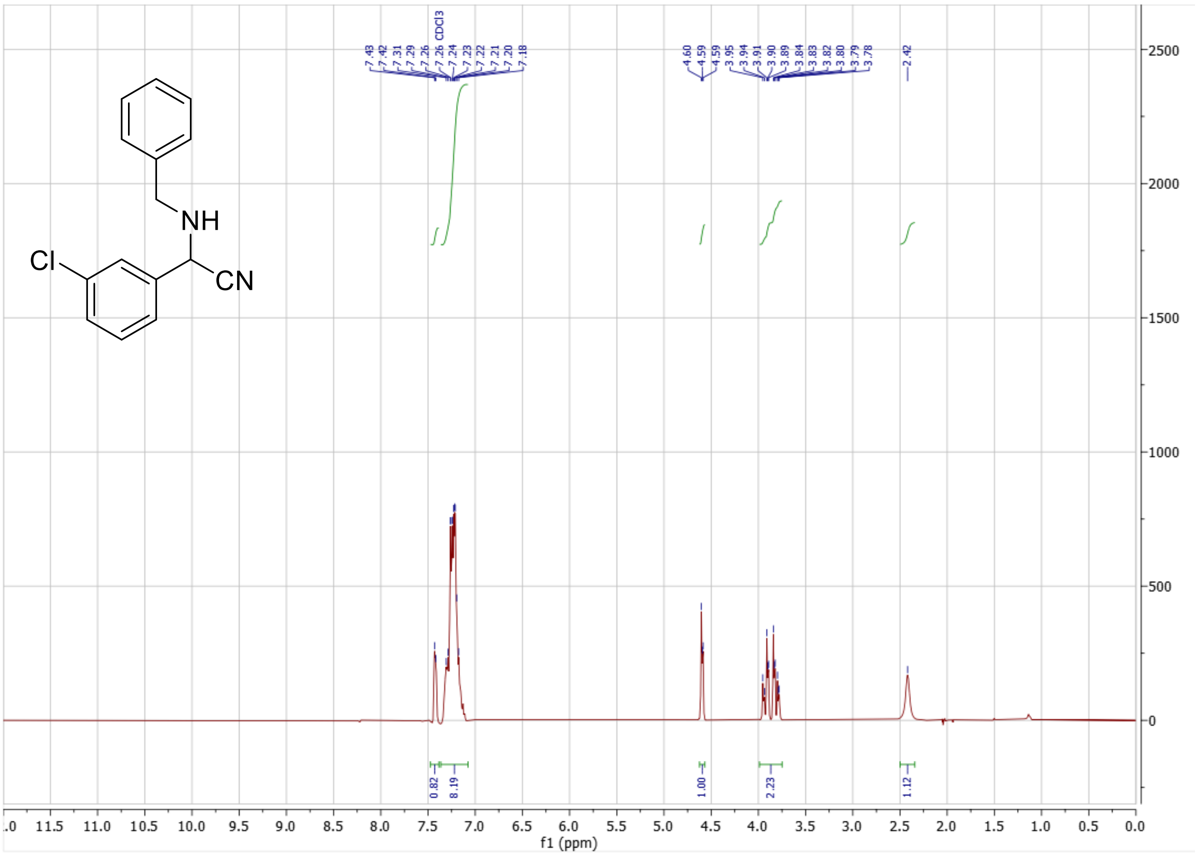

2-(Benzylamino)-2-(4-chlorophenyl)acetonitrile (**4ka**)

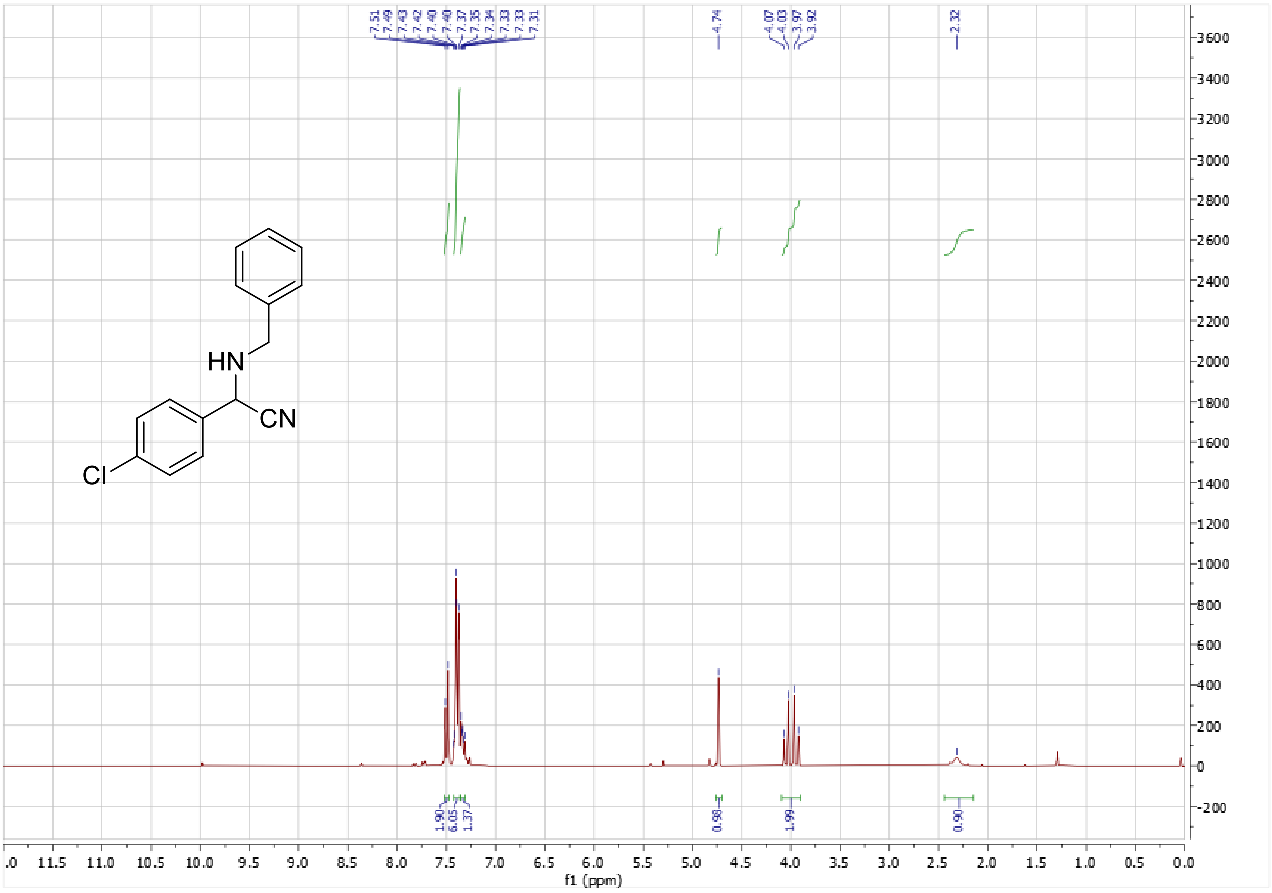

2-(Benzylamino)-2-(3-methylphenyl)acetonitrile (**4la**)

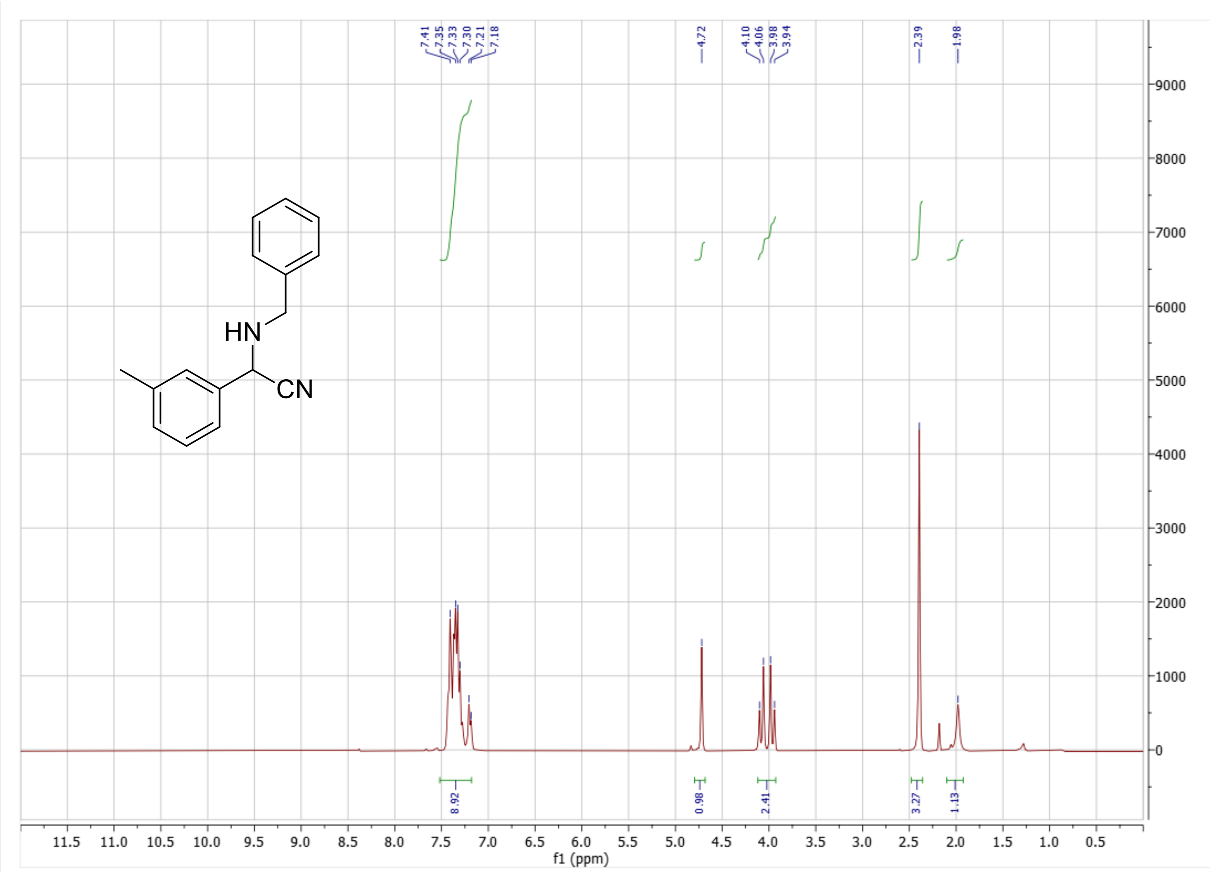

2-(Benzylamino)-2-(4-methylphenyl)acetonitrile (**4ma**)

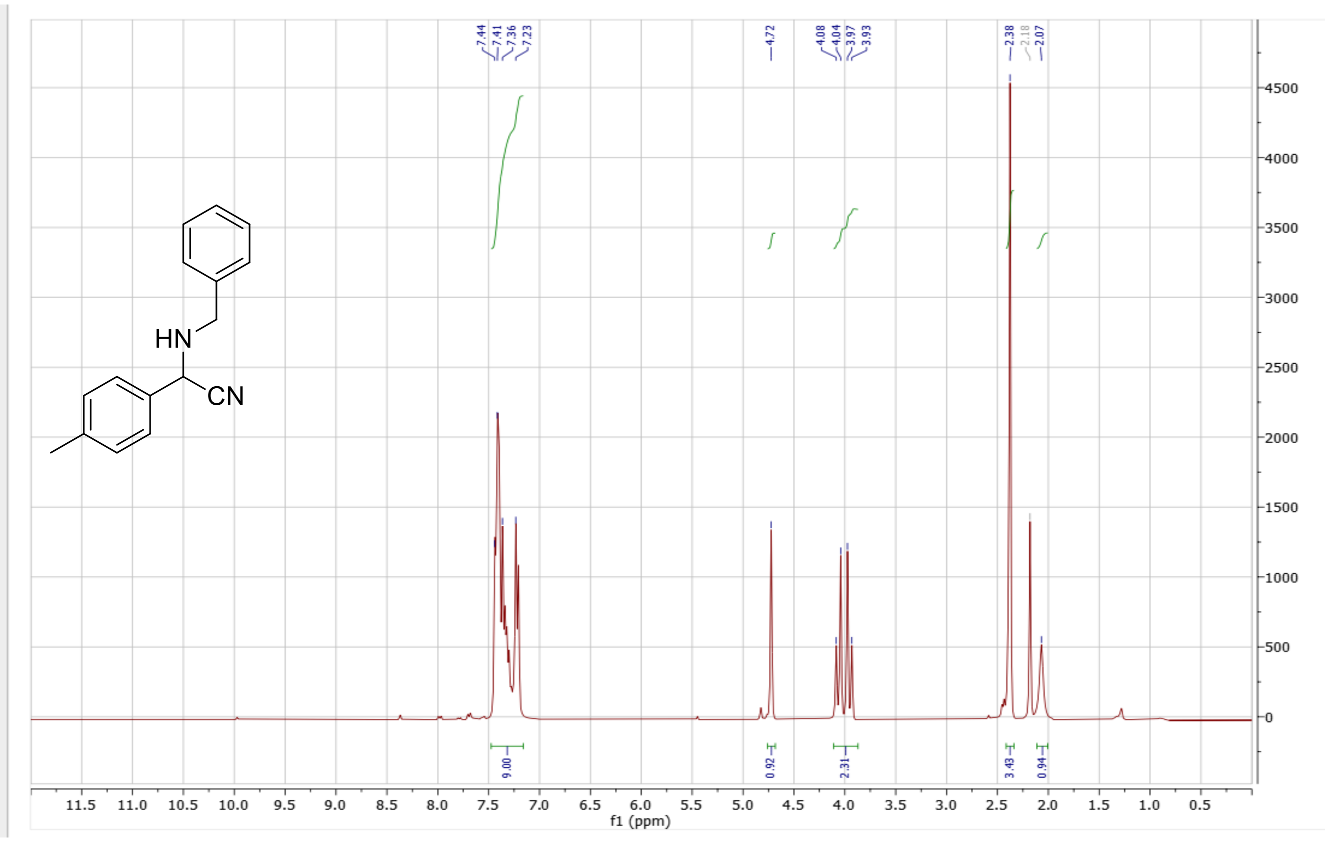

2-(Benzylamino)-2-(2-methoxyphenyl)acetonitrile (**4na**)

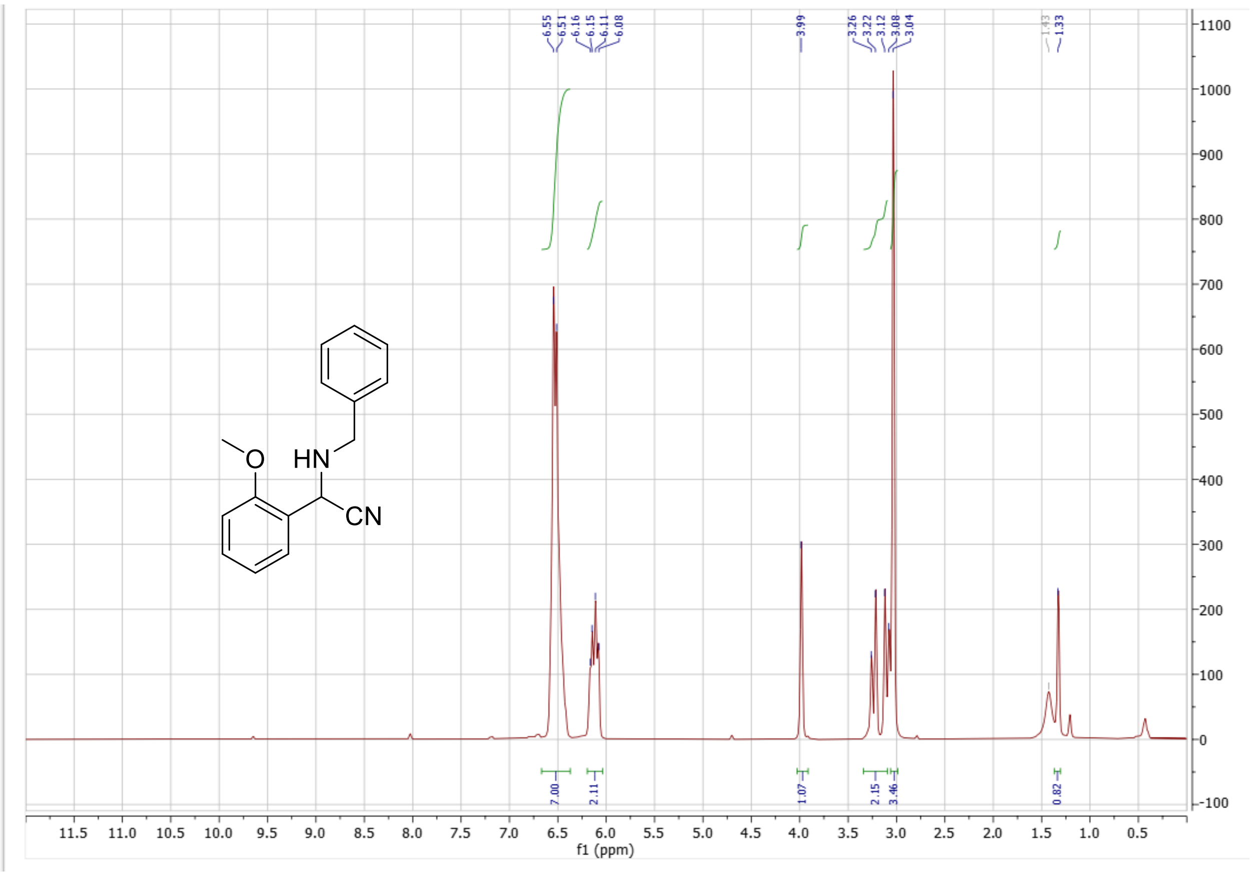

2-(Benzylamino)-2-(4-methoxyphenyl)acetonitrile (**4oa**)

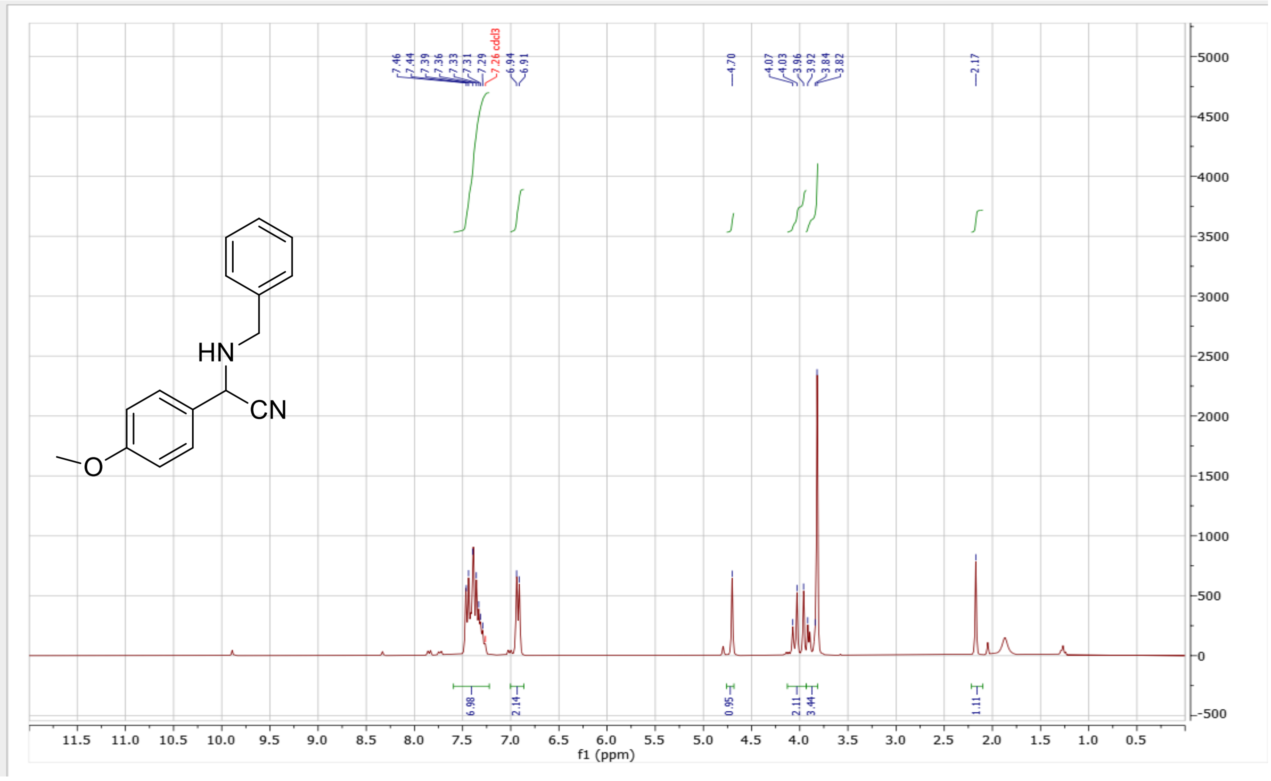

4-((Benzylamino)(cyano)methyl)benzonitrile (**4pa**)

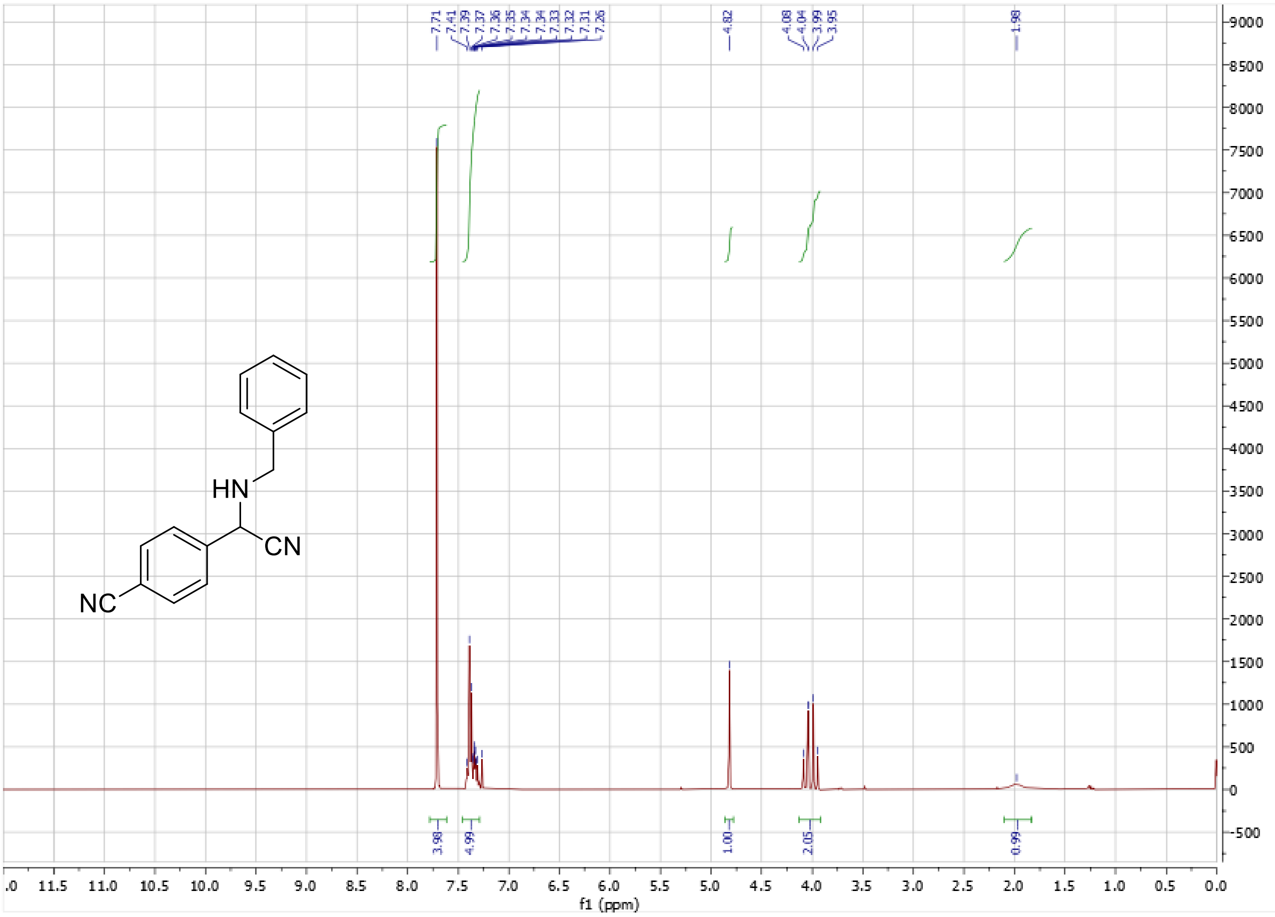

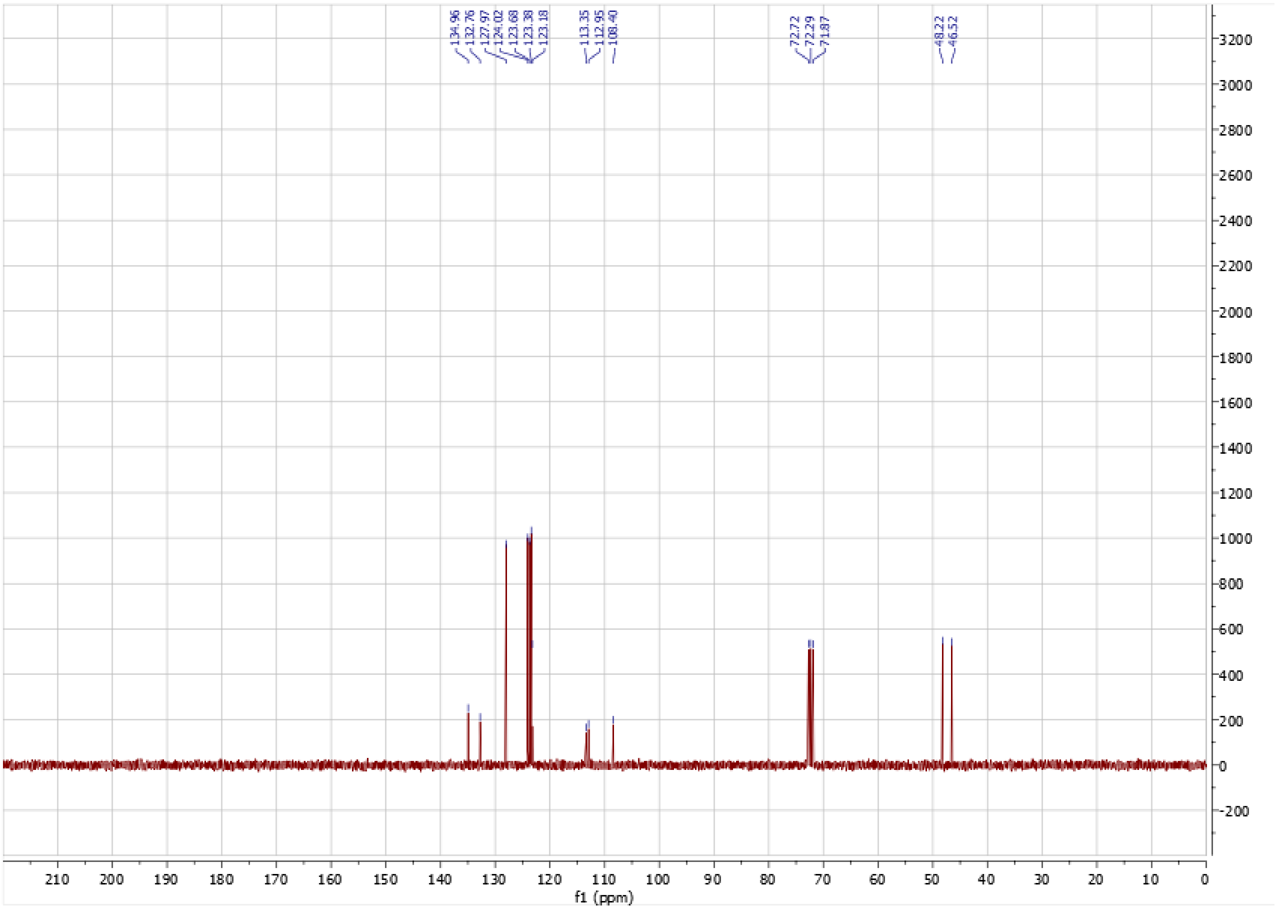

Supplement: Supplementary file 1 — Supplementary Material [file OPEN-14-e202500389-s001.pdf]
